# Correction of symptoms of Huntington disease by genistein through FOXO3-mediated autophagy stimulation

**DOI:** 10.1080/15548627.2023.2286116

**Published:** 2023-11-22

**Authors:** Karolina Pierzynowska, Magdalena Podlacha, Lidia Gaffke, Estera Rintz, Karolina Wiśniewska, Zuzanna Cyske, Grzegorz Węgrzyn

**Affiliations:** Department of Molecular Biology, Faculty of Biology, University of Gdańsk, Gdańsk, Poland

**Keywords:** Autophagy activation, correction of neurodegenerative disease symptoms, FOXO3, huntington disease mouse model, genistein (4′,5,7-trihydroxyisoflavone)

## Abstract

Huntington disease (HD) is a neurodegenerative disorder caused by a mutation in the *HTT* gene. The expansion of CAG triplets leads to the appearance of misfolded HTT (huntingtin) forming aggregates and leading to impairment of neuronal functions. Here we demonstrate that stimulation of macroautophagy/autophagy by genistein (4′,5,7-trihydroxyisoflavone or 5,7-dihydroxy-3-(4-hydroxyphenyl)-4 H-1-benzopyran-4-one) caused a reduction of levels of mutated HTT in brains of HD mice and correction of their behavior as assessed in a battery of cognitive, anxiety and motor tests, even if the compound was administered after symptoms had developed in the animals. Biochemical and immunological parameters were also improved in HD mice. Studies on molecular mechanisms of genistein-mediated stimulation of autophagy in HD cells indicated the involvement of the FOXO3-related pathway. In conclusion, treatment with genistein stimulates the autophagy process in the brains of HD mice, leading to correction of symptoms of HD, suggesting that it might be considered as a potential drug for this disease. Combined with a very recently published report indicating that impaired autophagy may be a major cause of neurodegenerative changes, these results may indicate the way to the development of effective therapeutic approaches for different neurodegenerative diseases by testing compounds (or possibly combinations of compounds) capable of stimulating autophagy and/or unblocking this process.

**Abbreviations**: CNS: central nervous system; EPM: elevated plus-maze; GOT1/ASPAT: glutamic-oxaloacetic transaminase 1, soluble; GPT/ALAT/ALT: glutamic pyruvic transaminase, soluble; HD: Huntington disease; HTT: huntingtin; IL: interleukin; mHTT: mutant huntingtin; NOR: novel object recognition; MWM: Morris water maze; OF: open field; ROS: reactive oxygen species; TNF: tumor necrosis factor

## Introduction

Huntington disease (HD) is a genetically transmitted, progressive neurodegenerative disease, inherited in an autosomal dominant manner [1]. It is caused by a mutation resulting in the expansion of CAG nucleotide triplets in exon 1 of the *HTT/IT15* (huntingtin) gene. The mutation results in the appearance of a long tract of glutamine residues in the HTT protein which impairs its proper folding. Thus, mutant HTT (mHTT) accumulates in cells, including neurons, as insoluble and hardly removable aggregates which impair the functions of these cells. Apart from mHTT aggregates, unproper secondary structures of mRNA molecules derived from the mutant *HTT* gene may disturb crucial cellular processes, like RNA splicing [2]. Then, both protein aggregates and faulty splicing can make an expression of many genes dysregulated [3], causing severe pathogenic effects in cells. These include dysfunction of lysosomes, impaired biogenesis of mitochondria, disrupted functions of organelles and improper protein transport, induction of inflammation, and enhanced oxidative stress [1]. At the organismal level, such pathological changes result in psycho-motoric dysfunctions, like changed behavior and impaired cognitive abilities. The quality of life of patients deteriorates over time, and they become dependent on their caregivers. Death usually occurs between 15 and 20 years after diagnosis [1].

Despite many attempts, no cure is still available for HD. Among therapeutic strategies considered to date, those based on either silencing the expression of the mutant gene or elimination of mHTT aggregates appear the most promising [4–6]. However, there are serious challenges in both these strategies. First, it is difficult to deliver therapeutic inhibitors of gene expression, such as anti-sense RNAs, into cells of the central nervous system (CNS), and then, it is problematic to silence only the mutant *HTT* allele while leaving the functional allele fully active. Second, the elimination of insoluble protein aggregates from the cells, especially neurons, is a complicated task, especially as too effective activation of protein-degrading cellular systems might result in severe adverse effects [7].

In light of the latter option, it is worth noting that impairment of the macroautophagy/autophagy process, which is responsible for the decay of improper and unwanted molecules or structures and damaged or dysfunctional organelles, has been demonstrated in HD [8–14]. Therefore, one might propose that stimulation of autophagy might be considered a therapeutic strategy for HD, and perhaps also other neurodegenerative diseases caused by the accumulation of misfolded and aggregated proteins. Indeed, such proposals have been published previously [7,15,16]. However, the major problem in such an approach is that the potential drug should have several properties. Namely, (i) it should effectively induce autophagy but at the same time it should not overstimulate this process, to avoid auto-destruction of cellular components, especially in the process of autophagy-dependent apoptosis; (ii) it should effectively cross the blood-brain-barrier to be easily delivered to CNS following oral (preferably) or intravenous administration; (iii) it should be safe and reveal little or no adverse effects in a long-time treatment, as such therapy of any genetic and/or neurodegenerative disease is expected to be lifelong [7]. In addition, in the case of HD, a disease in which enhanced oxidative stress and induction of inflammation are important secondary mechanisms leading to neurodegeneration [1], a therapeutic compound should ideally also reveal antioxidant and anti-inflammatory properties.

Although reduction of mHTT accumulation in animal and cellular models of HD in response to autophagy stimulation was first demonstrated some two decades ago [17,18], compounds like rapamycin (a strong autophagy inducer), express serious adverse effects when used for a longer time (despite they are registered as drugs for a short-time use in different purposes), including thrombocytopenia, anemia and leukopenia. Therefore, we were looking for compounds that are effective but gentle and safe autophagy stimulators, being also antioxidants and anti-inflammatory compounds. Our preliminary experiments suggested that genistein (4′,5,7-trihydroxyisoflavone or 5,7-dihydroxy-3-(4-hydroxyphenyl)-4 H–1-benzopyran-4-one) might be such a molecule. This isoflavone has already been known for acting as an antioxidant and an anti-inflammatory agent [19], however, we have demonstrated that it also activated autophagy in a cellular HD model, leading to the disappearance of mHTT aggregates [20]. These results were confirmed in experiments with the cells (fibroblast lines) derived from HD patients [21]. Moreover, genistein has been demonstrated to be safe for patients even at doses as high as 150 mg/kg/day administered orally for 1–2 years [22]. Therefore, in this study, we aimed to test the efficacy of genistein in the mouse model of HD (the R6/1 mice) by assessing the efficacy of this compound in the correction of behavioral (cognitive, anxiety, and memory) changes, biochemical abnormalities, oxidative stress enhancement, and induced inflammation. Unlike most other studies on the therapeutic effects of potential anti-HD drugs, we tested the effects when genistein was started to be administered after the appearance of serious symptoms in the animals to assess therapeutic rather than prophylactic efficacy. Moreover, molecular studies were performed (using both animal and cellular models) to learn abound principles of the mechanisms by which genistein stimulates autophagy in HD cells. To our knowledge, this is the first study demonstrating in a comprehensive manner that genistein stimulates autophagy through the FOXO3-related mechanism, thus, reducing levels of mHTT in the brains and correcting behavior of the HD mice as assessed in a battery of cognitive, anxious and motoric tests, even if administered after the animals have developed symptoms, and at the same time abolishing oxidative stress, inflammation, and stress responses. Therefore, we suggest that genistein can be considered as a putative drug for HD and that further studies in this direction are substantiated.

## Results

### The animal model of HD and the experimental schedule

To investigate the efficacy of genistein in the reduction of HD symptoms, the R6/1 mouse model of this disease has been employed (see the Materials and Methods section for a detailed description of this model). HD and control (wild-type, C57BL/6J) mice were studied, and behavioral, immunological and biochemical analyses were conducted at three time points. The first (initial) measurement was performed at the age of 8–9 weeks when no evident disease symptoms were observed. The second measurement was performed at the age of 18 weeks, when R6/1 mice experience significant deficits in spatial and short-term memory, and hyperactivity and the first motor disturbances begin to become apparent. At this stage of the disease, histological changes include gradual atrophy of some brain structures, including the striatum and hippocampus. The third measurement was performed at the age of 26 weeks, when the HD animals showed a significant decrease in body weight and an exacerbation of previously observed motor and cognitive impairments. The onset of treatment with genistein (oral administration of the dose of 150 mg/kg/day in the volume of 0.1 ml of water suspension) or water (oral administration of 0.1 ml of water; control experiments) was at the age of 16 weeks when the HD symptoms have already developed. Euthanasia was conducted at the age of 34–36 weeks, and biological material for biochemical and molecular investigations was collected. The scheme of the experiments is shown in Figure 1.

**Figure 1.**
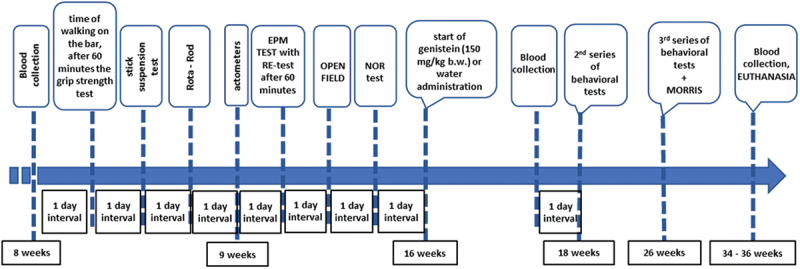
The scheme of the experiments with HD (R6/1) and control (C57BL/6J) mice.

### Correction of cognitive functions in HD mice by genistein

To test the effects of genistein on cognitive deficits in HD mice, a battery of behavioral tests was employed. The elevated plus-maze (EPM) test was dedicated to analyze anxious behavior and anxiety-related memory deficits. By recording the transfer latency from open (aversive) to closed (safe) arms of EPM, and including an additional measurement (re-test), it was possible to analyze memory processes. As indicated in Figure 2A, the transfer latency time became significantly longer in untreated and water-treated HD mice relative to that in control (healthy) mice during the test and re-test. However, treatment with genistein improved the results in HD mice, shortening the transfer latency times (in test and re-test) to a value statistically indistinguishable from those measured in experiments with control animals (Figure 2A). These results strongly suggested that anxiety-related memory deficits could be corrected by genistein in HD animals. Measurements of other memory-oriented parameters in the EPM test confirmed this suggestion (Figure S1).

**Figure 2.**
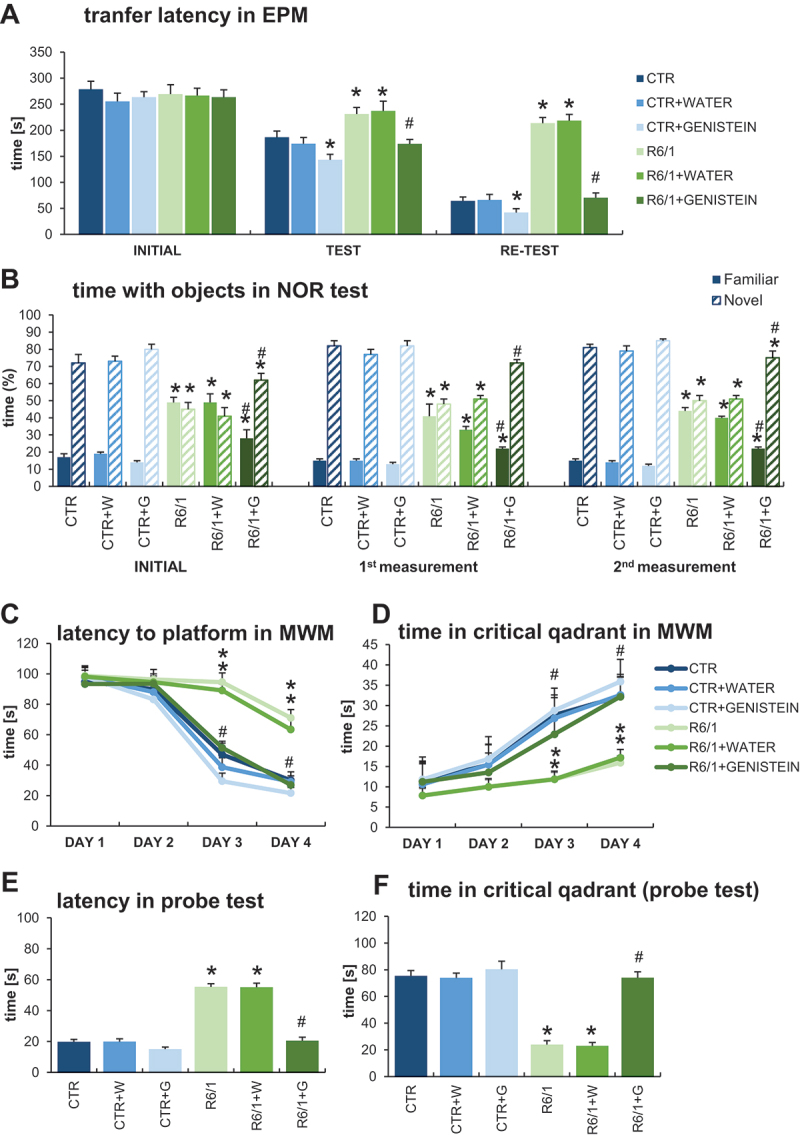
Correction of cognitive impairments in HD mice by genistein. HD mice (the R6/1 model) or control animals (the C57BL/6J line) were either untreated, treated with water, or treated with genistein (at the final dose of 150 mg/kg/day), starting from the age of 16 weeks. Various tests were performed with mice at the age of 9, 18 and 26 weeks (marked as INITIAL, 1^st^ measurement, and 2^nd^ measurement, respectively). Results obtained in elevated plus-maze (EPM) test (A), novel object recognition (NOR) test (B), and Morris water maze (MWM) test (C-F, with indicated type of measurement) are shown as mean values from measurements performed with 6 mice in each group with error bars indicating SD. Statistically significant differences (at *p* < 0.05) relative to untreated control (CTR) mice and HD mice (the R6/1 line) are indicated by asterisks and hashtags, respectively.

The novel object recognition (NOR) test was used to estimate working memory, attention, and anxiety. The results of measuring the time spent with objects in the NOR test demonstrated a significant abnormality in this parameter occurring in HD mice in comparison to control animals (Figure 2B). However, treatment with genistein significantly improved this feature, with a strong tendency toward the values measured in wild-type mice. Therefore, we conclude that genistein, administered after the appearance of the disease symptoms, can considerably improve the working memory and attention in HD mice.

The Morris water maze (MWM) test is one of the most widely used tests to assess spatial memory. The learning capabilities of mice were assessed in this test to indicate that HD animals reveal severe spatial memory deficits, as assessed in measuring the latency to the platform (Figure 2C), time spent in critical quadrant (Figure 2D), latency in the probe test (Figure 2E), and time spent in critical quadrant determined in the probe test (Figure 2F). Quite unexpectedly, genistein corrected the spatial memory deficits in HD mice, as estimated by measuring the above-mentioned parameters in the MWM test (Figure 2 C-E). The results obtained with genistein-treated HD mice were statistically undistinguishable from those determined in control animals. More parameters were also measured in the MWM test (as shown in Figure S2) and these results corroborated the above presented conclusion. Importantly, the speed of swimming of HD mice did not differ significantly from that of control animals (Figure S2), indicating that the observed differences in finding the platform in the MWM test were due to cognitive impairment occurring in untreated and water-treated HD mice rather than physical disability of these animals.

### Improvement/Normalization of anxiety behavior and motor deficits in HD mice by genistein

To assess anxiety behavior, we have performed several tests designated to estimate specific anxiety parameters. In the EPM test, HD mice spent significantly shorter time in the open arms of EPM (Figure 3A), and significantly longer time in the closed arms (Figure 3B) than control mice. Moreover, time spent in the center of EPM was drastically shorter in the experiments with HD mice than in the case of wild-type mice (Figure 3C). These results confirmed the strong abnormalities in anxiety behavior occurring in the HD mouse model. However, treatment with genistein caused total correction of these abnormalities in HD mice, as the values measured in this group reached the levels determined in control animals (Figure 3 A-C). Again, more measurements of the EPM test parameters confirmed this conclusion (Figure S1).

**Figure 3.**
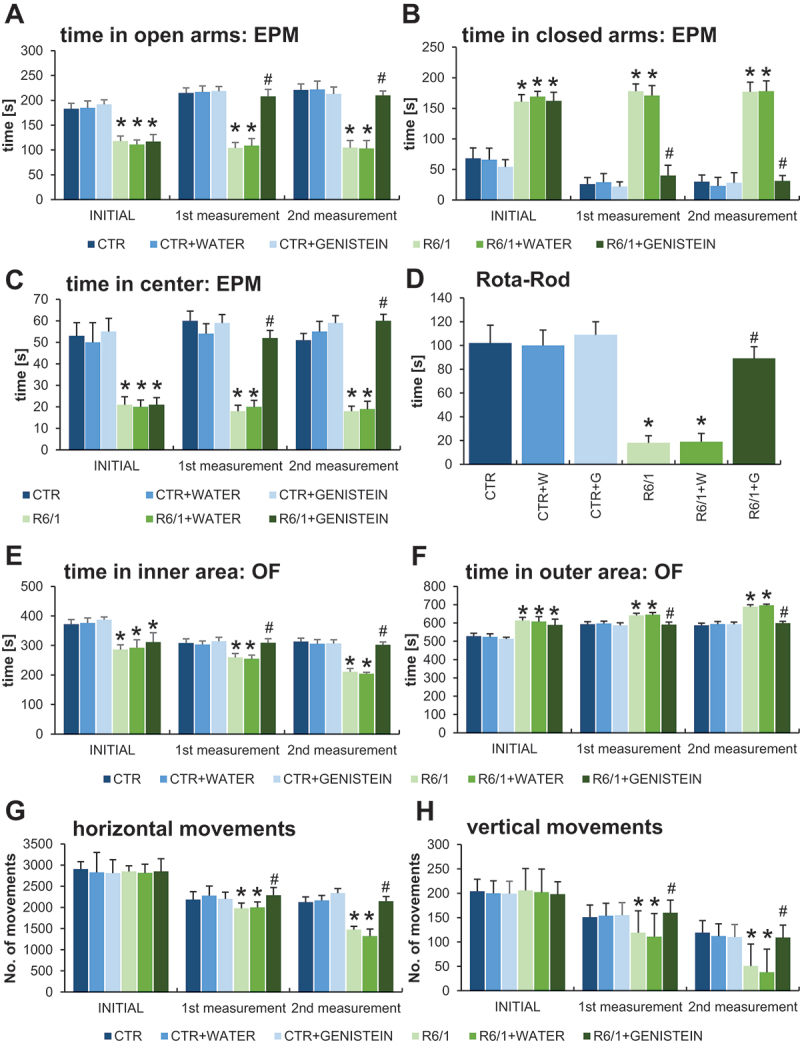
Improvement/Normalization of anxiety behavior and motor deficits in HD mice by genistein. HD mice (the R6/1 model) or control animals (the C57BL/6J line) were either untreated, treated with water, or treated with genistein (at the final dose of 150 mg/kg/day), starting from the age of 16 weeks. Various tests were performed with mice at the age of 9, 18 and 26 weeks (marked as INITIAL, 1^st^ measurement, and 2^nd^ measurement, respectively). Results obtained in elevated plus-maze (EPM) test (A-C, with the indicated type of measurement), rota-rod test (D), open field (OF) test (E-F, with the indicated type of measurement), and actometer (G-H, with the indicated type of measurement) are shown as mean values from measurements performed with 6 mice in each group with error bars indicating SD. Statistically significant differences (at *p* < 0.05) relative to untreated control (CTR) mice and HD mice (the R6/1 line) are indicated by asterisks and hashtags, respectively.

Interpretation of the results of the open field (OF) experiments corroborated the conclusion made on the basis of the EPM test. Namely, in the OF test, HD animals spent less time in the inner area (central squares) (Figure 3E) and less time in the outer area (peripheral squares) (Figure 3F) than wild-type mice. Again, treatment with genistein significantly improved this anxiety behavioral abnormality, as the results of genistein-treated HD mice were evidently changed toward those obtained with the control groups (Figure 3 E-F). Confirmation of this interpretation of the results was demonstrated by other measurements performed in the OF test (Figure S3) which also corroborated the conclusion on the improvement of cognitive functions in genistein-treated HD mice (Figure S4).

Since motor coordination is severely affected in both HD patients and the R6/1 mouse model of HD, this parameter was assessed using the rota-rod test. As expected, drastically impaired motor coordination was evident in HD mice in this test, however, this deficit could be significantly improved by administration of genistein (Figure 3D). Motor deficits were also observed in the experiments performed with the use of an actometer. The number of horizontal (Figure 3G) and vertical (Figure 3H) movements was significantly lower in HD mice relative to control animals, while treatment with genistein again improved these parameters considerably. The same conclusion could be reached when analyzing ambulatory movements (Figure S5).

Motor disability in HD mice was also evidenced by performing the wire handling test (Figure 4) and by measuring grip strength using a computerized grip force meter (Figure 5). Again, HD mice treated with genistein revealed a significant improvement in motor skills.

**Figure 4.**
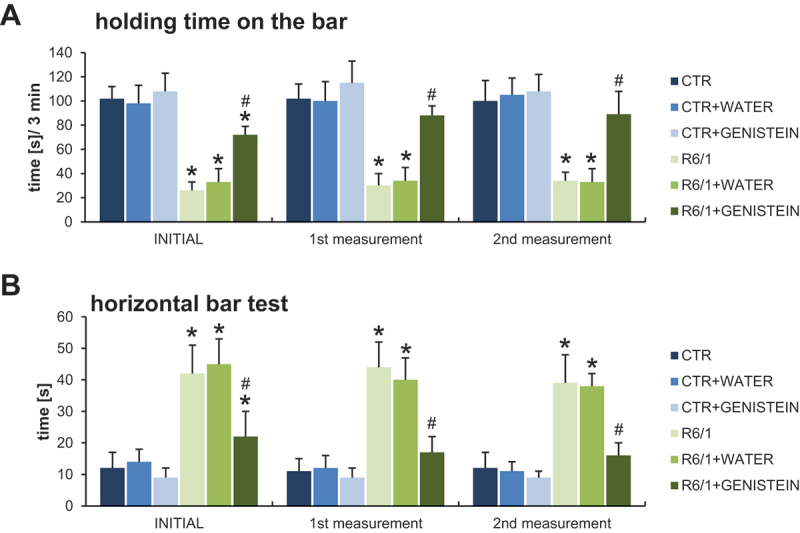
Motor parameters in HD mice treated with genistein as assessed in the wire handling test. HD mice (the R6/1 model) or control animals (the C57BL/6J line) were either untreated, treated with water, or treated with genistein (at the final dose of 150 mg/kg/day), starting from the age of 16 weeks. The tests were performed with mice at the age of 9, 18 and 26 weeks (marked as INITIAL, 1^st^ measurement, and 2^nd^ measurement, respectively). Results are shown as mean values from measurements performed with 6 mice in each group with error bars indicating SD. Statistically significant differences (at *p* < 0.05) relative to untreated control (CTR) mice and HD mice (the R6/1 line) are indicated by asterisks and hashtags, respectively.

**Figure 5.**
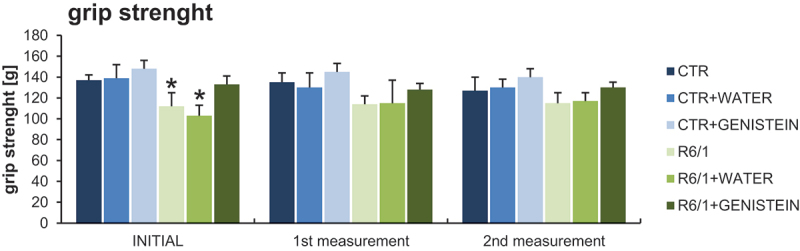
Grip strength measurement in HD mice by treated with genistein. HD mice (the R6/1 model) or control animals (the C57BL/6J line) were either untreated, treated with water, or treated with genistein (at the final dose of 150 mg/kg/day), starting from the age of 16 weeks. The tests were performed with mice at the age of 9, 18 and 26 weeks (marked as INITIAL, 1^st^ measurement, and 2^nd^ measurement, respectively). Results are shown as mean values from measurements performed with 6 mice in each group with error bars indicating SD. Statistically significant differences (at *p* < 0.05) relative to untreated control (CTR) mice and HD mice (the R6/1 line) are indicated by asterisks and hashtags, respectively.

### Normalization of inflammatory processes by genistein in HD mice

By measurements of levels of pro-inflammatory and anti-inflammatory cytokines in the blood, we have confirmed that the inflammation process is induced in HD mice. Namely, levels of IL1B/IL-1β and TNF/TNFα were considerably increased in HD animals relative to the control mice, while IL6 and IL10 revealed an opposite tendency (Figure 6 A-D). However, levels of all these cytokines were normalized in HD mice treated with genistein. The levels of the stress and pro-inflammatory hormone – corticosterone have been increased in the HD mouse model relative to controls while being normalized in genistein-treated animals (Figure 6E). Therefore, we conclude that inflammatory response is induced in HD mice, but the levels of cytokines are normalized by the treatment with genistein.

**Figure 6.**
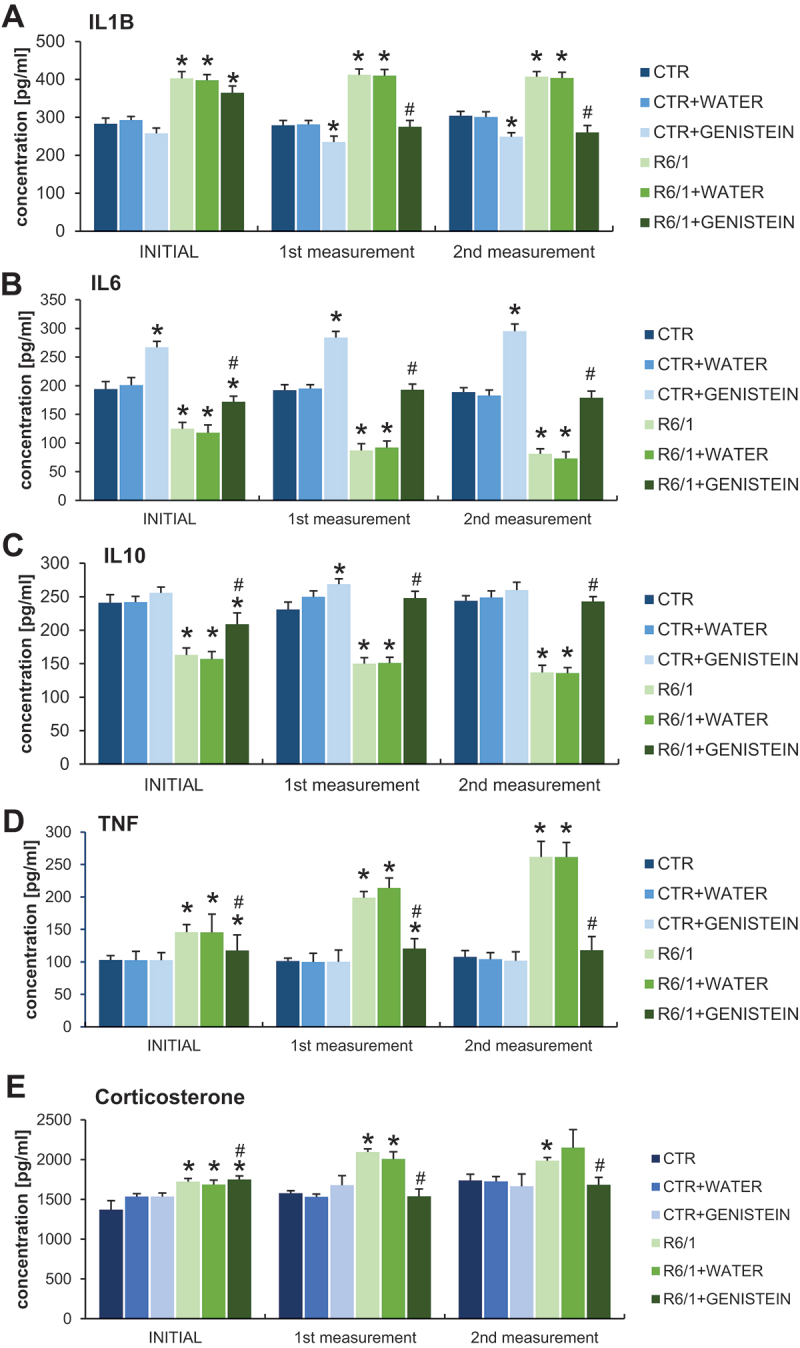
Normalization of inflammatory processes by genistein in HD mice. HD mice (the R6/1 model) or control animals (the C57BL/6J line) were either untreated, treated with water, or treated with genistein (at the final dose of 150 mg/kg/day), starting from the age of 16 weeks. Levels of IL1B (A), IL6 (B), IL10 (C), TNF (D), and corticosterone (E) were measured. Statistically significant differences (at *p* < 0.05) relative to untreated control (CTR) mice and HD mice (the R6/1 line) are indicated by asterisks and hashtags, respectively.

### Effects of genistein on selected hematological and biochemical parameters in blood, skeletal muscles, and heard muscle of HD mice

Apart from the cognitive, anxiety behavior, motor, and inflammation regulation abnormalities in HD animals, we have also noticed that many other parameters are changed in R6/1 mice. Especially, the biochemical parameters in blood, skeletal muscles, and heard muscle (Figure 7) as well as hematological parameters were changed in HD mice relative to controls. However, significant improvement in hematological parameters in HD animals was observed after treatment with genistein (Figures S6-S8). Moreover, positive effects of such treatment were evident for the above-mentioned biochemical parameters, especially in levels or activities of GPT/ALAT, GOT1/ASPAT, bilirubin, CK (creatine kinase), TNN (troponin I), and reactive oxygen species (ROS) (Figure 7).

**Figure 7.**
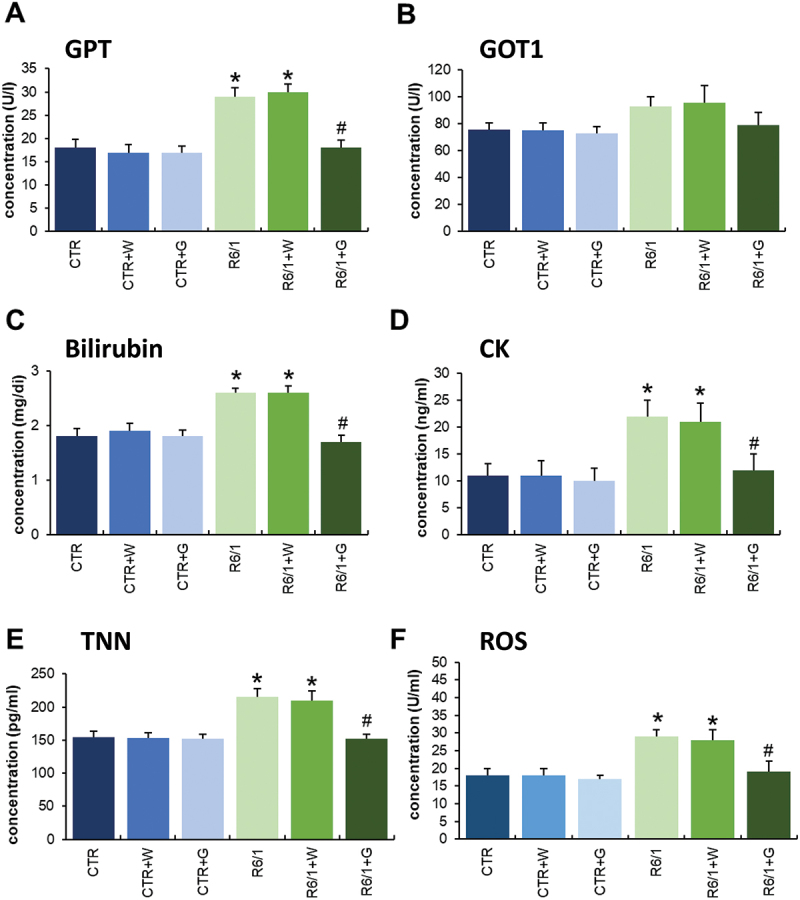
Biochemical parameters measured in HD mice treated with genistein. HD mice (the R6/1 model) or control animals (the C57BL/6J line) were either untreated, treated with water, or treated with genistein (at the final dose of 150 mg/kg/day), starting from the age of 16 weeks. Results are shown as mean values from measurements performed with 6 mice in each group with error bars indicating SD. Statistically significant differences (at *p* < 0.05) relative to untreated control (CTR) mice and HD mice (the R6/1 line) are indicated by asterisks and hashtags, respectively.

### Reduction of mHTT aggregates by genistein treatment in the brains of HD mice

We have demonstrated previously that treatment with genistein caused a significant reduction of levels of mHTT aggregates in a cellular model of HD [20] and in fibroblasts derived from HD patients [21]. Therefore, in the light of correction of behavioral abnormalities, motor dysfunctions, and biochemical and immunological changes in HD mice (the R6/1 model), we have tested whether similar effects of genistein treatment can be observed in the brains of HD animals after oral administration of genistein at the dose of 150 mg/kg/day. The presence of aggregates of HTT was tested in selected brain structures, including the striatum, cerebellum, and cortex. The presence of the aggregates was confirmed in cells of all these brain structures in HD mice, contrary to the healthy controls (Figure 8; note that the aggregates are stuck at the top of the gel or capillary during the electrophoresis, irrespective of the actual molecular mass of the protein [20,21]). However, treatment with genistein led to the elimination of these HTT structures from the brain tissue (Figure 8, Figure S9). These results indicate that similarly to the cellular model of HD and patient-derived fibroblasts, mHTT aggregates disappear also in the brains of animals after treatment with genistein.

**Figure 8.**
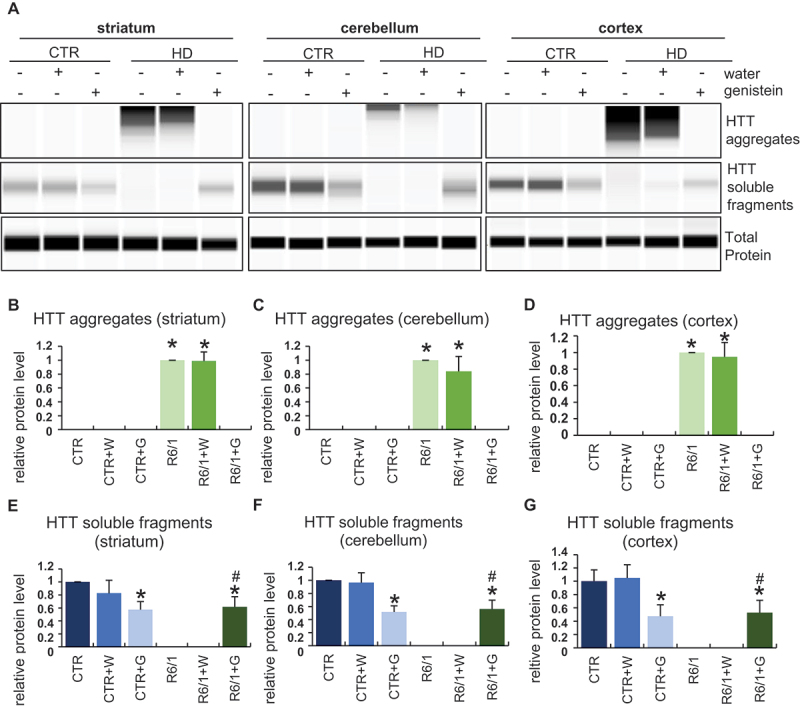
Reduction of levels of HTT aggregates in the brains of HD mice treated with genistein. HD mice (the R6/1 model) or control animals (the C57BL/6J line) were either untreated, treated with water, or treated with genistein (at the final dose of 150 mg/kg/day), starting from the age of 16 weeks. Levels of HTT were measured in the striatum, cerebellum, and cortex of the brains of mice at the age of 36 weeks. Panel A shows representative western blots. Panels B-G represent quantification of the results, shown as mean values from measurements performed with 6 mice in each group with error bars indicating SD. HTT levels were normalized to the total protein amount. Statistically significant differences (at *p* < 0.05) relative to untreated control (CTR) mice and HD mice (the R6/1 line) are indicated by asterisks and hashtags, respectively.

The levels of the soluble form of wild-type HTT were also to some extent reduced in control mice treated with genistein, though a significant amount of this protein remained unaffected (Figure 8). This indicates that a part of HTT molecules can be degraded under conditions of the genistein administration, though this process is of relatively low efficiency. Interestingly, soluble HTT levels were drastically decreased in untreated and water-treated HD mice, while this form of the protein was as abundant in genistein-treated R6/1 mice as in genistein-treated wild-type animals (Figure 8). One should note that R6/1 mice contain the human exon 1 with multiple CAG repeats inserted into their genomes, however, they have also the intact wild-type *HTT* gene (see Materials and Methods for the model description and references). Therefore, the soluble HTT form represents the wild-type protein. One might suggest that the wild-type HTT protein can be trapped by mHTT in the aggregates, while genistein-dependent elimination of these structures could liberate normal HTT, occurring again in the soluble form (as indicated above, HTT aggregates are stuck during the electrophoresis at the top of the capillary, thus their migration is halted irrespective of the actual molecular weight of polypeptide(s) forming them).

### Genistein-mediated autophagy stimulation by genistein in the brains of HD mice

Since the reduction of mHTT levels by genistein was demonstrated by us previously to be dependent on the induction of autophagy in the HD cellular model [20], we asked whether such a phenomenon occurs also in the brains of HD mice. Hence, levels of autophagy markers were measured in selected brain structures of such mice, as well as control animals. The level of the LC3-II protein, the most widely used autophagy marker, was significantly increased in the striatum, cerebellum, and cortex of the brains of genistein-treated wild-type and HD mice, strongly suggesting that this isoflavone stimulates the autophagy process there (Figure 9A-D). Such a conclusion was corroborated by results of measuring levels of the SQSTM1/p62 protein which dropped in cells with induced autophagy by genistein (Figure 9E). Stimulation of autophagy correlates with upregulation of TFEB (transcription factor EB), a transcription factor responsible for stimulation of lysosome biogenesis. Therefore, positive regulation of TFEB in the brains of genistein-treated wild-type and HD animals (Figure 9E-H) provided another proof for the efficient stimulation of the autophagy process in the brain by genistein. Interestingly, we have observed the appearance of TFEB-mHTT aggregates in the striatum of HD mice, but not in other brain regions, and not in control mice (Figure 9 9,I-9). Such aggregates disappeared after oral administration of genistein to animals.

**Figure 9.**
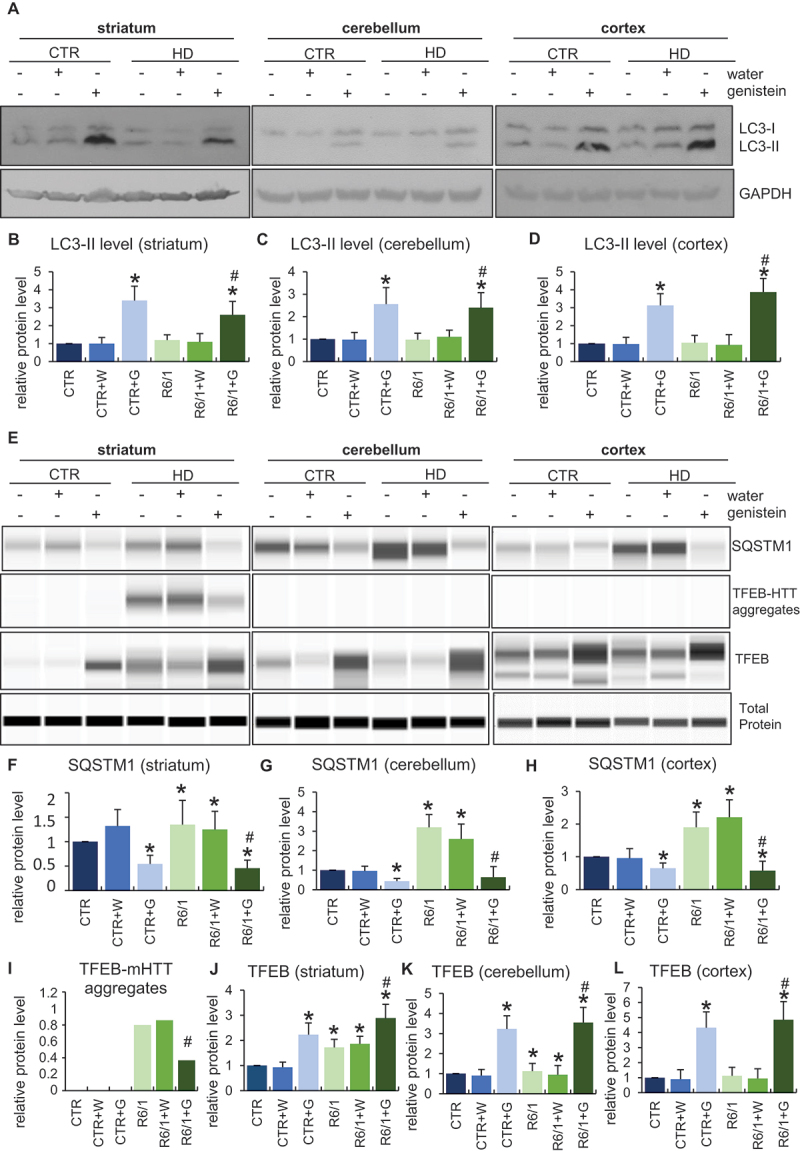
Induction of autophagy by genistein in the brains of HD mice treated with genistein. HD mice (the R6/1 model) or control animals (the C57BL/6J line) were either untreated, treated with water, or treated with genistein (at the final dose of 150 mg/kg/day), starting from the age of 16 weeks. Levels of selected autophagy-related marker proteins were measured in the striatum, cerebellum, and cortex of the brains of mice at the age of 36 weeks. Panels A and E show representative western blots. Panels B-D and F-L represent quantification of the results, shown as mean values from measurements performed with 6 mice in each group with error bars indicating SD. Levels or investigated proteins were normalized to the total protein amount. Statistically significant differences (at *p* < 0.05) relative to untreated control (CTR) mice and HD mice (the R6/1 line) are indicated by asterisks and hashtags, respectively.

### The mechanism of autophagy stimulation by genistein in HD

To learn more about the stimulation of the autophagy process in HD, we used patient-derived fibroblast in molecular cell biology experiments. As indicated previously [20,21], we confirmed that autophagy is activated in these cells by treatment with genistein, as assessed by an increase in the levels of the LC3-II protein (Figure 10F-G). As expected, such induction occurred also in control fibroblasts (Figure 10 10-G). To learn about the mechanism of genistein-mediated autophagy stimulation, we have measured levels of RPS6KB/S6K and EIF4EBP1 proteins, substrates of the MTOR kinase (an inhibitor of the autophagy process), as well as levels of the phosphorylated forms of these substrate proteins. We found that these levels were significantly decreased in cells treated with genistein, and the dose-response correlation was evident (Figure 10 10-E), suggesting that the kinase activity of MTOR was inhibited, leading to autophagy stimulation. The strong induction of lysosomal biogenesis and autophagy under these conditions has been confirmed by monitoring the abundance of lysosomes, as assessed by using the LysoTracker Red and microscopic analyses. These experiments evidenced a considerable increase in the abundance of lysosomes in genistein-treated HD and control cells (Figure 10 10-I). Similar effects were observed also in the brain cells of animals treated with genistein (Figure 11).

**Figure 10.**
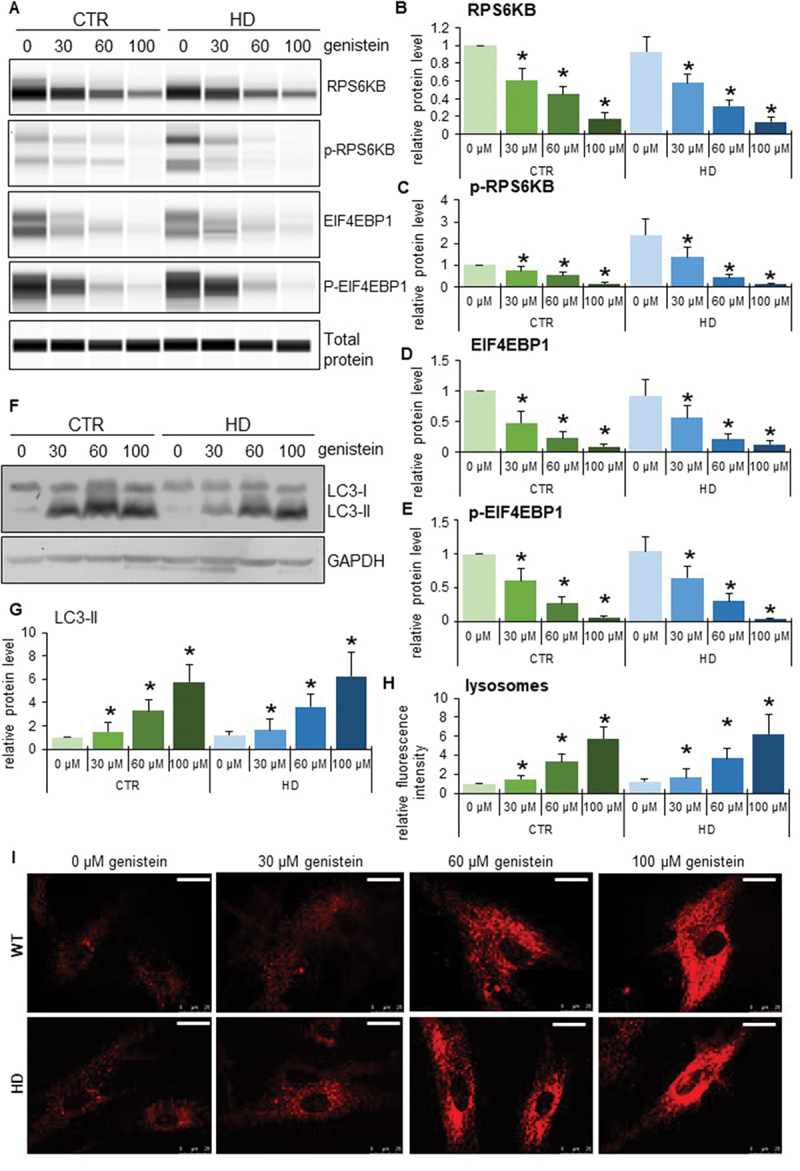
The pathway of autophagy stimulation by genistein in fibroblasts derived from HD patients. Fibroblasts derived from HD patients (HD) or control, healthy persons (CTR) were cultured and treated with either 0.1% DMSO or genistein (at the final concentration of 30, 60 or 100 µM) for 24 h. Representative western blots are shown in panels A and F, and quantification of levels of RPS6KB/S6K, phosphorylated RPS6KB/S6K, EIF4EBP1, phosphorylated EIF4EBP1, and LC3-II is shown in panels B, C, D, E, and G, respectively. The abundance of lysosomes in cells either untreated or treated with genistein, as visualized by LysoTracker Red staining, is shown in panel I, and the results were quantified as indicated in panel H. Panels B-E, G, and I represent mean values from measurements performed with 4 cell lines in each group with error bars indicating SD. Levels of LC3-II were normalized to the GAPDH amount, while levels of other investigated proteins were normalized to the total protein amount. Bars in panel I: 25 µm. Statistically significant differences (at *p* < 0.05) relative to untreated control (CTR) fibroblasts and HD cells (HD) are indicated by asterisks and hashtags, respectively.

**Figure 11.**
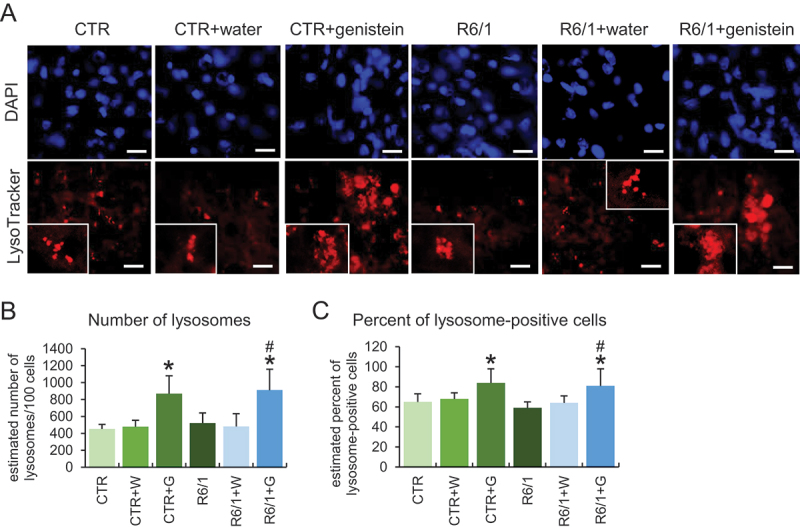
Effect of genistein on lysosome abundance in HD mice. The abundance of lysosomes was estimated in cells of the brains of C57BL/6J (control; CTR) and R6/1 (HD model) either untreated, treated with water, or treated with genistein (at the final dose of 150 mg/kg/day), starting from the age of 16 weeks and analyzed at the age of 36 weeks. Lysosomes were visualized by LysoTracker Red staining while nuclei were stained with DAPI. Representative photographs obtained in a fluorescence microscope are shown in panel A (bars: 25 µm), and the results were quantified as indicated in panels B (average number of lysosomes) and C (percent of lysosome-positive cells). Statistically significant differences (at *p* < 0.05) relative to untreated controls (CTR) and HD mice (HD) are indicated by asterisks and hashtags, respectively.

To test whether genistein-mediated effects on HD are related to autophagy, we have employed fibroblasts derived from patients and healthy controls. One should note that in human HD fibroblasts, there are two forms of HTT, normal and mutant, both produced as effects of expression of corresponding alleles of the *HTT* gene. As large proteins, these forms are undistinguishable in their migration during electrophoresis and are equally well recognized by anti-HTT antibodies. Therefore, we have measured total levels of HTT, according to the previously established and validated procedure [21]. We found that inhibition of autophagy by chloroquine resulted in impairment of the genistein-dependent reduction of HTT levels in cells (Figure 12). Therefore, we conclude that genistein improves the cellular phenotype of HD by stimulation of autophagy.

**Figure 12.**
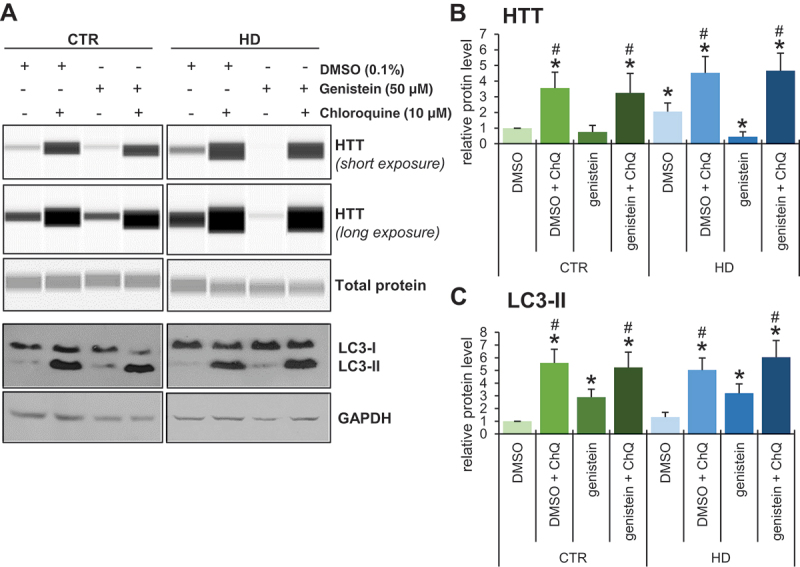
Impairment of genistein-mediated effects on levels of HTT by chloroquine-induced autophagy inhibition in fibroblasts derived from HD patients and control subjects. Fibroblasts derived from HD patients (HD) or control, healthy persons (CTR) were cultured and treated with 0.1% DMSO, 50 µM genistein, 10 µM chloroquine or a combination of genistein (50 µM) and chloroquine (50 µM) for 24 h. Representative western blots are shown in panel A, and quantification of levels of HTT and LC3-II is shown in panels B and C, respectively. Each group (CTR and HD) is represented by 4 cell lines. Levels of LC3-II were normalized to the GAPDH amount, while HTT levels were normalized to the total protein amount. Statistically significant differences (at *p* < 0.05) relative to DMSO-treated control (WT) cells and HD fibroblasts are indicated by asterisks and hashtags, respectively.

To find which specific transcription factor mediates the autophagy induction by genistein in HD, we performed a series of experiments to find that levels and phosphorylation of the FOXO3 protein were significantly decreased upon treatment with genistein. This was evident in both selected brain regions of mice (striatum, cerebellum, and cortex) (Figure 13 13-G) and in cultures of HD fibroblasts (Figure 13 13-I). Moreover, the translocation of FOXO3 from the cytoplasm to the cell nucleus was evident in genistein-treated fibroblasts (Figure 14). These phenomena were not restricted to HD cells and animals but occurred also in controls. Therefore, one might propose that genistein indirectly activates the FOXO3 transcription factor which stimulates the expression of genes involved in autophagy.

**Figure 13.**
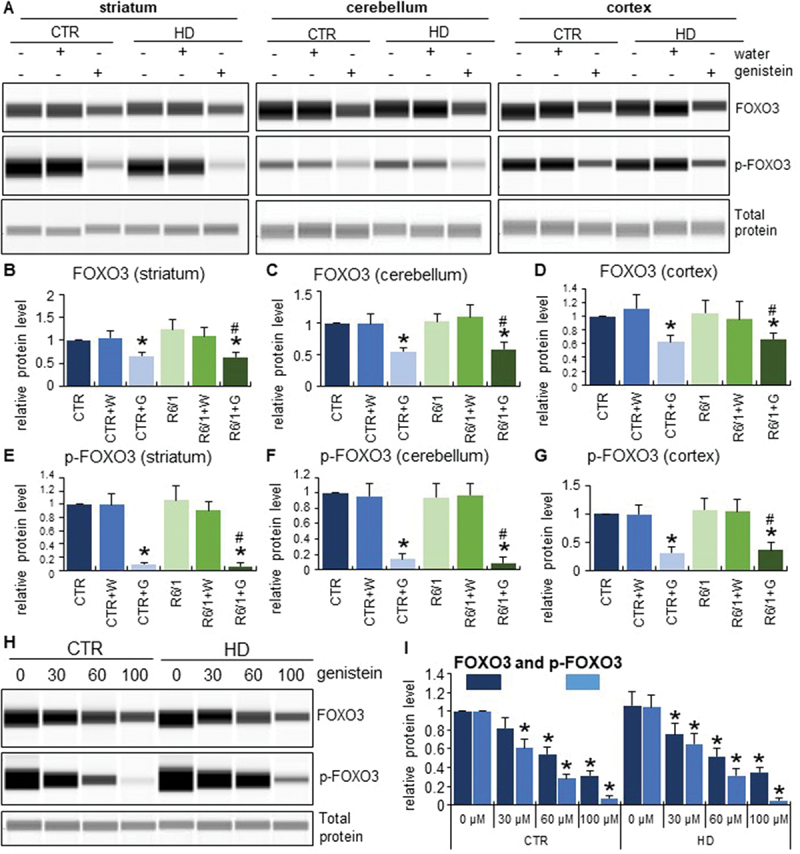
Activation of FOXO3 by genistein in the brains of HD mice and patient-derived HD fibroblasts. HD mice (the R6/1 model) or control animals (the C57BL/6J line) were either untreated, treated with water, or treated with genistein (at the final dose of 150 mg/kg/day), starting from the age of 16 weeks. Fibroblasts derived from HD patients (HD) or control, healthy persons (CTR) were cultured and treated with either 0.1% DMSO or genistein (at the final concentration of 30, 60 or 100 µM) for 24 h. Levels of FOXO3 and its phosphorylated form (p-FOXO3) were measured in the striatum, cerebellum, and cortex of the brains of mice at the age of 36 weeks, and in fibroblasts treated with either genistein or 0.1% DMSO for 24 h. Panels A and H show representative western blots. Panels B-G and I represent the quantification of the results, shown as mean values from measurements performed with either 6 mice in each group (panels B-G) or 4 cell lines in each group (panel I), with error bars indicating SD. Levels of the investigated proteins were normalized to the total protein amount. Statistically significant differences (at *p* < 0.05) relative to untreated control (CTR) mice or cells and HD mice (the R6/1 line) or cells are indicated by asterisks and hashtags, respectively.

**Figure 14.**
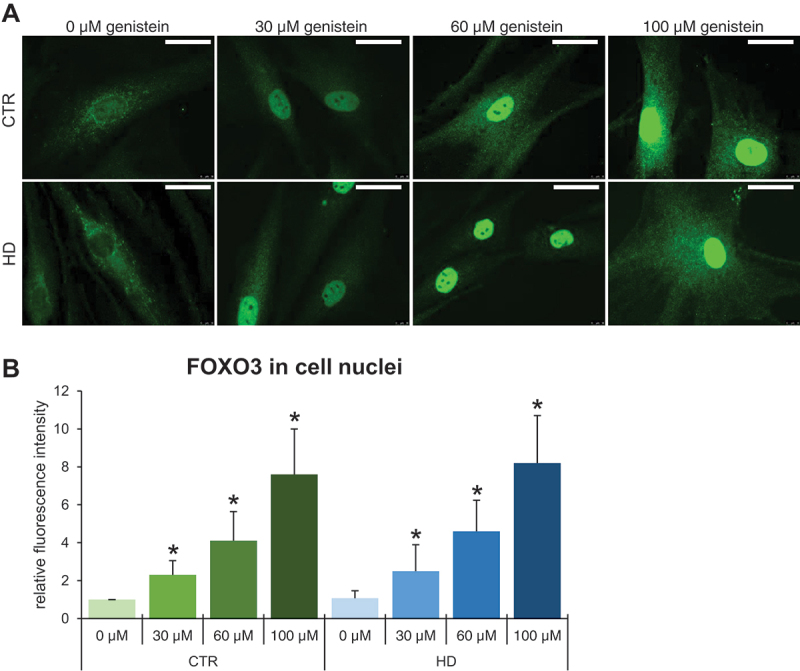
Translocation of FOXO3 from the cytoplasm to the nucleus in HD and control cells treated with genistein. Fibroblasts derived from HD patients (HD) or control, healthy persons (CTR) were cultured and treated with either 0.1% DMSO or genistein (at the final concentration of 30, 60 or 100 µM) for 24 h. Translocation of FOXO3 from the cytoplasm to the nucleus in cell culture experiments was observed by fluorescence microscopy (A), and quantification of the signals was conducted by measurement of fluorescence intensity in 100 randomly chosen cells from each sample (B). Bars in panel A: 25 µm. Statistically significant differences (at *p* < 0.05) relative to untreated control (CTR) cells or HD cells are indicated in panel B by asterisks or hashtags, respectively.

To test the hypothesis that FOXO3 is involved in the genistein-mediated stimulation of autophagy and subsequent elimination of mHTT aggregates, we have silenced the expression of the FOXO3-encoding gene in human fibroblasts derived from both HD patients and control individuals and monitored the effects of genistein on HTT levels. We found that while HHT levels were higher in HD fibroblasts relative to control cells, most probably reflecting the presence of the undegraded mutant form apart from the soluble fraction, genistein treatment resulted in the levels comparable to those observed in normal fibroblasts (Figure 15). However, silencing of expression of *FOXO3* by specific siRNAs resulted in impairing the efficiency of the genistein-mediated decrease in HTT levels (Figure 15). These results corroborated the proposal that genistein can stimulate the autophagy process (which is required to degrade the accumulated mHTT protein) at least partially through activation of the FOXO3 factor.

**Figure 15.**
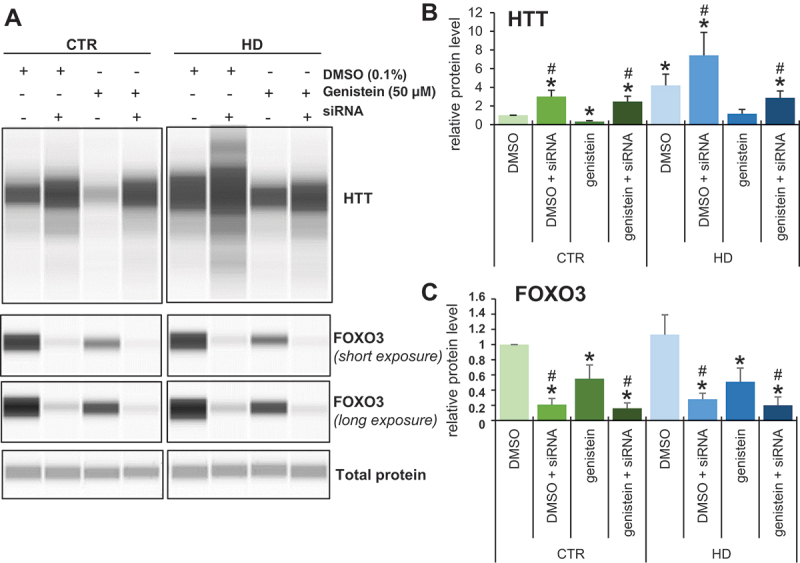
Impairment of genistein-mediated effects on levels of HTT by siRNA-mediated silencing of the expression of the *FOXO3* gene in fibroblasts derived from HD patients and control subjects. Fibroblasts derived from HD patients (HD) or control, healthy persons (CTR) were cultured and treated with either 0.1% DMSO or 50 µM genistein for 24 h. In addition, the *FOXO3* gene expression was silenced using specific siRNA (added 48 h before addition of either DMSO or genistein), where indicated. Representative western blots are shown in panel A, while quantification of levels of HTT and FOXO3 is shown in panels B and C, respectively. Each group (CTR and HD) is represented by 4 cell lines. Levels of HTT and FOXO3 were normalized to the total protein amount. Statistically significant differences (at *p* < 0.05) relative to DMSO-treated control (CTR) cells and HD fibroblasts are indicated by asterisks and hashtags, respectively.

## Discussion

The severity of HD and the lack of effective treatment have led to efforts to develop an effective therapy for this disease substantiated, important and urgent [1,4–6]. As a genetic, neurodegenerative disease, it is, however, especially difficult to treat. Since the major problem in HD pathogenesis is the formation of insoluble aggregates of the product of the mutated *HTT* gene, one of the potential therapeutic options is to decrease the levels of the toxic protein [4–7]. To achieve this, it is necessary to activate a cellular process capable of degrading protein aggregates, and autophagy meets such a requirement. Therefore, autophagy stimulation was proposed as a therapeutic option for HD [7,15]. On the other hand, a candidate for an anti-HD drug should not only activate the autophagy process effectively, but at the same time, it should be safe (not causing significant adverse effects) in a lifelong treatment, cross the blood-brain-barrier, and optimally also act as an antioxidant and anti-inflammatory agent to diminish secondary, though still important pathological mechanisms of the disease [1,7]. Our previous preliminary studies suggested that genistein, one of the isoflavones, may meet all these features [20,21]. Indeed, this compound, apart from the stimulation of the autophagy process, reveals many biological activities, including anti-inflammatory, antioxidant, anti-angiogenic, anti-obesity, anti-cancer, cardioprotective, and anti-diabetic properties [19]. Therefore, in this study, we aimed to perform a comprehensive study on the effects of this compound administration in the HD mouse model. Unlike many other studies on searching for anti-HD therapy, the onset of administration of the tested compound was after the development of the disease symptoms rather than before such a disease stage. This allowed us to determine whether genistein treatment can correct the already disturbed functions of the organism, not only to prevent or delay the appearance of the specific symptoms.

The presented results demonstrate that treatment with genistein at the dose of 150 mg/kg/day not only improved the behavior of HD mice, but even corrected most of the tested parameters to levels undistinguishable from the control (healthy) animals. Since a battery of tests measuring cognitive functions and anxiety was employed, and in all types of assays the significant improvement in HD animals was evident, it can be concluded that genistein corrects behavior in this model. Moreover, motoric functions which are severely impaired in HD (also in the R6/1 mouse model of HD) could be corrected by daily oral genistein administration. Importantly, various biochemical and hematological parameters of the blood of HD mice were considerably changed relative to control animals, whereas genistein treatment corrected these abnormalities. Similar to them, levels of oxidative stress in muscles normalized, as did levels of pro- and anti-inflammatory interleukins in the blood of HD animals. All these results suggest that the efficacy of genistein in the correction of so many HD symptoms, even when the administration of this compound was started after the appearance of the abnormalities in animals, may be due to several biological activities and complex action of this isoflavone.

One should note that while the neurodegenerative aspects of HD are crucial in this disorder, other changes play also a significant role in the development of various symptoms. Although the nature of IL6 (pro-inflammatory or anti-inflammatory) is constantly discussed in the literature, it plays an important role in HD. It has a role not only in the immune system response but, due to its central expression, also in the course of cognitive processes. Changes in IL6 levels in HD patients are the earliest described markers of impaired immune response, appearing up to 16 years before the onset of motor dysfunction [23]. It was demonstrated that in the R6/2 HD mouse model with IL6 deficiency, there was a dysregulation of many genes related to synaptic function, as well as the BDNF receptor NTRK2 and disruption of the behavioral pattern [23]. Moreover, at early stages of HD, elevated IL6 levels are thought to have protective effects, similar to those in animal models of traumatic brain injury, Parkinson and Alzheimer diseases. However, as the disease progresses, when the number of mHTT deposits increases, resulting in serious abnormalities in the functioning of the immune system, there is a decrease in IL6 levels, not only in peripheral blood but also in its central expression, which translates into the severity of various categories of pathological disturbances. Elevated levels of anti-inflammatory cytokines, such as IL4 and IL10, were observed in the plasma of HD patients only at the pre-symptomatic stage of the disease or when the symptoms were still mild. IL10 plays an important role in the course of many different diseases, being responsible primarily for supporting the repair processes of tissues whose structure has been damaged as a result of infection or inflammation, as well as for suppressing the pro-inflammatory reaction, which ultimately leads to the above-mentioned damage. At the advanced stage of Huntington disease, decreased levels are observed not only in the blood plasma but also in the central nervous system, indicating impaired inflammation-suppressing processes, as well as weakening of repair mechanisms [24]. In this light, the normalization of levels of factors involved in the immune response by treatment with genistein indicates that this isoflavone might efficiently control not only the primary but also secondary pathomechanisms of HD.

The above conclusion can be corroborated by the improvement of various biochemical parameters in HD mice by genistein, including activities of GPT/ALAT (glutamic pyruvic transaminase, soluble) and GOT1/ASPAT (glutamic-oxaloacetic transaminase 1, soluble), and levels of bilirubin, CK (creatine kinase), TNN (troponin I), and ROS. These biochemical markers were determined in view of monitoring changes in the functioning of organs, such as the liver, kidneys, and heart, whose gradual dysfunction is observed in HD [25].

Despite other genistein biological activities, autophagy stimulation appears to be the major one in its anti-HD effectivity. We have proven that this stimulation is effective in both HD mouse models and human cells. Activated autophagy could lead to the efficient degradation of already accumulated mHTT, and a significant reduction of levels of this mutant protein in both mouse brains and human fibroblasts. Therefore, we have asked what the molecular mechanism of genistein-mediated activation of autophagy is. Previous studies indicated that genistein stimulated TFEB, which is the master regulator of lysosomal biogenesis [26]. Nevertheless, there are at least 7 pathways of autophagy stimulation, thus, it was important to learn more details about the genistein’s activity in this process. Especially important is also the fact that one of the recent reports indicated that mHTT co-aggregates with TFEB [27], making the attempts to stimulate autophagy solely through activation of this transcription factor doubtful. In this study we demonstrate that genistein, apart from influencing levels and modifications of known general autophagy markers, significantly modulated both levels and phosphorylation of FOXO3, resulting in the translocation of this transcription factor to the nucleus. Importantly, silencing of the *FOXO3* gene expression impaired the effect of genistein on the reduction of the mHTT levels, supporting the proposal that genistein-mediated stimulation of autophagy, which is responsible for the effective removal of mHTT aggregates, requires FOXO3. Indeed, recent studies by others have shown that FOXO3 positively regulates the expression of genes involved in autophagy, stimulating this process effectively [28,29]. One might speculate that such a regulation could positively influence the course of neurodegenerative disease. Although the role of this transcription regulator in various neurodegenerative diseases has been discussed recently, it was rather considered a factor implicated in the control of aging and longevity [30]. However, since FOXO3 may activate the expression of autophagy-related genes [28,29], it could also prevent neurodegeneration through the reduction of toxic aggregates in cells. Our results corroborate such suggestions while showing for the first time that genistein is a stimulator of FOXO3.

Previous attempts to stimulate autophagy in order to reduce the levels of mHTT involved the use of various compounds. The earliest works were based on the treatment with rapamycin, a strong inhibitor of MTOR [17,18]. However, this compound, although registered now as a drug, reveals serious adverse effects when used for a longer period. Felodipine, a calcium channel blocker, used currently as an anti-hypertensive drug, was shown to stimulate autophagy which prevented the accumulation of misfolded proteins [31], though its ability to reduce already accumulated aggregates remains unknown. In another study, autophagy was stimulated by overexpression of the gene coding for either TFEB or Beclin-1, but the effects on mHTT aggregates were limited [32]. CGAS (cyclic GMP-AMP synthase) was shown to activate both inflammatory and autophagy responses [33] which was an interesting discovery, however, it would have perhaps limited applications in searching for a potential anti-HD drug as inflammation stimulation would not be desirable in this case. Some authors proposed that the MTOR-RPS6KB-EIF4EBP signal transduction pathway might be a target for an anti-HD drug based on autophagy stimulation [34], though further studies are required to demonstrate the efficacy of such a potential therapeutic option, and the role of AMPK in autophagy induction cannot be underestimated in this case [35]. The GSK3 protein is another regulator of autophagy, and the use of a selective inhibitor of GSK3, called L807mts, resulted in a decrease in the amounts of mHTT aggregates in HD cells [36]. Treatment of the R6/2 mouse model with L807mts indicated the neuroprotective effect and improvement of symptoms, though total correction could not be demonstrated [36]. Deletion of the SUMO1 was suggested as a possible target for anti-HD drugs as deletion of the corresponding gene in the mouse model, as well as the use of a SUMOylation inhibitor in cell cultures, resulted in the autophagy stimulation and lower levels of mHTT [37]. Erythropoietin was then shown to modulate the PI3K-AKT-MTOR-RPS6KB pathway of autophagy stimulation and improvement in the rat model of HD induced by 3-nitropropionic acid [38]. However, in this model, only symptoms resemble those occurring in humans suffering from HD, while no HTT aggregates occur, therefore, it is difficult to estimate the effects of such a therapy when used in patients. It is also worth noting that regulation through microRNA was proposed to be important in autophagy stimulation in HD cells [39]. Finally, a recent report demonstrated that an artificial compound, called J3, stimulated autophagy, and when administered to HD mice showed a slight improvement in the motor functions in the open-field test, balance beam test, and rotarod tests [40]. Interestingly, in the same tests (open-field and rotarod) performed with HD mice treated with genistein in this work, total correction rather than slight improvement of symptoms was noted. These results might suggest a higher efficacy of genistein than J3 in the normalization of pathological changes caused by HD.

Normal HTT is required for basal autophagy, and defects in this process in HD cells were reported previously [6]. In this light, it is interesting that in the brains of R6/1 mice which express both transgenic human *HTT* exon 1 with multiple CAG repeats and the wild-type mouse *HTT* gene, the level of soluble HTT protein was significantly decreased. After treatment with genistein, soluble HTT became significantly more abundant, and occurred at a level similar to that found in wild-type mice which received genistein (this level was significantly, though not dramatically, lower than in non-treated control animals). This may suggest that normal HTT can be trapped by mHTT aggregates, thus being inactivated, and – between others – unable to stimulate autophagy. Such a scenario would mean a positive feedback regulation loop, leading to the formation of more and more aggregates. Genistein, through independent stimulation of the autophagy process, might restore the cellular machinery degrading macromolecular complexes, and thus, rescue the ability of cells to remove toxic aggregates.

Importantly, a very recent report demonstrated that the blockage of the autophagy process through stimulation of the MTOR activity, mediated by the activation of the CCR5 receptor (C-C motif chemokine receptor 5), might be a major cause of neurodegeneration [41]. These pathological processes may occur even before the appearance of disease symptoms, as demonstrated with the mouse model of HD [38]. A promising result was the finding that pharmacological inhibition of CCR5 improves the neuropathology in HD and tauopathy by unblocking the autophagy process, thus causing the removal of toxic aggregates [41]. That important work, together with results presented in this report, strongly suggested that either stimulating or unblocking autophagy might be a putative way to either prevent neurodegeneration or correct this pathological process or both. This may shed new light on the development of novel therapeutical strategies for neurodegenerative diseases.

In summary, treatment with genistein stimulates the autophagy process in the brains of HD mice, acting through the FOXO3 pathway. This leads to the correction of behavioral symptoms of HD. Moreover, oxidative stress and inflammatory processes are normalized. These results suggest that genistein might be considered a drug for HD. Considering these results, together with very recently published studies, showing that inhibition of the CCR5 receptor may unblock the impairment of autophagy [41], it is tempting to propose that activation of this process might be crucial in developing effective therapies for HD and some other neurodegenerative diseases. This might be achieved by optimization of the autophagy stimulation through an effective and safe compound (like genistein) or by direct activation of FOXO3 (overcoming the problem of a relatively low genistein bioavailability) or by using improved CCR5 inhibitors. A combination of the above-mentioned strategies of autophagy stimulation might also be considered.

## Study limitations

There are some limitations of this study. First, the R6/1 mouse model of HD consists of transgenic mice which express the exon 1 of the human *HTT* gene with extension of the CAG repeats, together with the native mouse *HTT* variant [42,43]. Although this model is useful in laboratory studies, and represents an accelerated form of the disease, it differs from the genetic configuration of human patients where one normal and one mutant (but bearing all exons and introns) variants of the *HTT* gene are expressed. Therefore, one should avoid to simply extrapolate putative effects of genistein in HD patients from the results presented in this report. Second, only males of the R6/1 model were investigated in this work, due to previously reported differences in the disease course between R6/1 males and females [44], and potential difficulties with interpretation of the results in the case of the latter group. However, HD occurs in humans with equal frequency in both males and females. Therefore, further studies employing mice of both sexes are necessary to fully understand effects of genistein on the disease and to estimate efficacy of this isoflavone in treatment of males and females.

## Materials and Methods

### Animals

Experiments were carried out with 18 males of the R6/1 line (B6.Cg-Tg (HDexon 1)61Gpb/J; Jackson Laboratories; USA) of mice, a widely used model of Huntington disease [42,43], while the control group consisted of 18 C57BL/6J male mice (Tri-City Central Animal Laboratory, Research and Service Center of the Medical University of Gdansk, Poland). The R6/1 model, which is characterized by a slower disease progression (though still significantly accelerated relative to the human disease) than in many other models, was chosen to allow a thorough investigation of the genistein therapeutic potential not only in the context of central nervous system changes but also peripheral pathophysiological disturbances resulting from the accumulation of mHTT aggregates in various organs and tissues. This model has recently been characterized in detail by our team in light of its use in such studies [44]. Employing numerous behavioral, immunological, and biochemical analyses, we were able to determine the critical time points at which specific groups of symptoms begin to develop in mice. This allowed us not only to analyze the progression of the disease but also to monitor the effectiveness of the therapy, which is often implemented when motor disturbances are evident, and the peripheral pathophysiological changes are already strongly aggravated. The selection of time points for analyses was based on our previous studies and literature data [44]. The use of males in the experiments was due to the specific aims of this study and the characteristic features of the chosen animal model. As demonstrated previously in the work devoted to characterizing the R6/1 model in detail, males and females differ significantly in many of the parameters, both in terms of immune response and behavioral pattern [44]. In addition, females show a significantly more aggressive course of the disease, manifested by a faster progression and severity of symptoms [44]. It has been suggested that the influence of sex hormones is crucial here, but as our study showed, the overactivity of the hypothalamic-pituitary-adrenal stress axis is also an important determinant of the above-mentioned differences [44]. Another aspect that must be taken into account when analyzing the results obtained with females is the proper synchronization of their hormonal cycle. If this factor is omitted, the results obtained might not be reliable. This requires additional modification of the entire experimental procedure. Considering such a large number of variables and fundamental differences in the course of the disease in R6/1 mice of both sexes, and the fact that we aimed to check whether genistein can be an effective therapeutic agent in the treatment of HD, we decided to conduct experiments only with males.

Behavioral, immunological, and biochemical analyses were conducted at three time points (Figure S1). The first (initial) measurement was taken when the mice were 8–9 weeks old, and no symptoms were observed. The second measurement was taken when the mice were 18 weeks old. After this time, R6/1 mice show significant deficits in spatial and short-term memory, develop hyperactivity and the first motor disturbances begin to become apparent [41]. Histological changes include gradual atrophy of some brain structures, like the striatum and hippocampus. The last analyses were performed when the mice were 26 weeks old. At this age, the R6/1 animals show a significant decrease in body weight and an exacerbation of previously observed motor and cognitive impairments.

Mice were housed in a ventilated animal room (15 air changes per hour). Stable conditions were maintained, including artificial lighting (12 h light − 12 h dark), ambient temperature (22 ± 2°C) and humidity (50 ± 5%), with access to food (with reduced isoflavone content) and tap water ad libitum. Mice were maintained in approved laboratory cages, 15-cm high and at least 400 cm^2^ in size. To ensure the most optimal enrichment of the environment, suitable attractants and accessories for rodents were used. The animal facility met the requirements of the Act on the Protection of Animals Used for Scientific or Educational Purposes, dated 15 January 2015 (Journal of Laws dated 26 February 2015), as well as the recommendations of the European Commission on the welfare of animals used in scientific experiments. All experiments were approved by the Local Ethics Committee for the Care and Use of Laboratory Animals in Bydgoszcz, Poland (resolution no. 17/2018).

### Experimental groups of mice

Animals were divided into 6 experimental groups, each with 6 individuals, (i) “C57BL/6J control group” (mice that did not receive water or genistein (Pharmaceutical Research Institute in Warsaw, 446-72-0), (ii) “C57BL/6J + water” (mice that received water in a volume of 0.1 ml daily), (iii) “C57BL/6J + genistein” (mice that received genistein at the dose of 150 mg/kg/day), (iv) “R6/1 control group” (Huntington disease model mice that were not supplemented with water or genistein), (v) “R6/1 + water” (mice that received water in a volume of 0.1 ml daily), (vi) “R6/1 + genistein” (mice that received genistein at a dose of 150 mg/kg/day). In groups “C57BL/6J + genistein” and “R6/1 + genistein”, genistein supplementation was started when the animals were 16 weeks old. In R6/1 mice, deterioration of memory processes, increased motor activity and anxiety, as well as the first motor disturbances are already evident after 16 weeks of life [41]. Genistein (suspended in water; note that genistein is hardly soluble in water) was administered daily at the dose of 150 mg/kg/day, orally in the volume of 0.1 ml, via a 25 mm-long metal gavage. This resulted in the entrance of the tip of the gavage into the stomach or a very distal esophagus, ensuring that the appropriate dose of the tested compound was given to the animal’s organism. The use of the gavage was necessary to ensure that the indicated dose was delivered to the mice. In control experiments, 0.1 ml of water was administered in the same way. Before administration of genistein or water, mice were subjected to a gavage habituation procedure for 14 days.

### The elevated plus-maze test (EPM)

The EPM is a test dedicated to the analysis of anxiety behavior in rodents. Open arms are an anxiety-generating aversive factor that can limit rodent’s natural tendency to explore a new environment. By recording the transfer latency from open (aversive) to closed (safe) arms, and including an additional measurement (re-test), it is also possible to analyze memory processes [41]. The experimental apparatus consisted of a cross-shaped platform, placed 50 cm above the ground. The EPM test is made of plexiglass and consists of two white open arms (50 cm x 10 cm) – aversive – evoking a sense of danger in the animals, and two black opposite arms, surrounded by 40 cm high walls (closed). A white square is located in the center (10 cm x 10 cm) to connect the two types of arms. Animal behavior was recorded using a camera connected to an automatic tracking system (EthoVision XT, Noldus, Netherlands). After each measurement, the platform was cleaned with 70% ethanol and allowed to dry for 5 min to eliminate the influence of odor traces on the next animal’s behavior. In each group, the test was carried out at the same time of day (between 8:00 and 12:00 a.m.). Registration was performed three times when mice reached 9, 18, and 26 weeks of age. Each assessment consisted of two measurements, lasting 5 min and separated by a 60 min interval (re-test). The test started by placing the mouse at one end of the open arm with its tail directed to the center. Registered reactions included the transfer latency (the transition time from the open (aversive) arm to the closed (safe) arm) reflecting memory processes, and the time spent in open and -closed arms of the maze (anxiety). The behavioral activity was scored every minute of the test in order to separate the learning/memory-related activity and fear/anxiety.

### The novel object recognition test (NOR)

The NOR test is used to measure working memory, attention, and anxiety, and to investigate the response to novelty in rodents. The experimental apparatus consisted of a square arena (100 cm x 100 cm) with a white floor divided by black lines into 25 equal squares, surrounded by white walls (60 cm high), and a set of three objects – two identical cups and one different, large wooden cube (difference in color, shape, and texture). The test was conducted from 8:00 to 12:00 a.m. Each measurement started when the mouse was placed in the corner of the area (starting square). Objects were placed in the middle of the arena, always in the same squares. Following each recording, the surface of the arena was cleaned with 70% ethanol and allowed to dry (5 min) to eliminate the effects of odor traces on the behavior of the mice. Animal activity was recorded using a video camera, connected to an automatic tracking system (EthoVision XT, Noldus, Netherlands).

The test was performed three times (when mice reached 9, 18, and 26 weeks of age) in three series according to the following scheme. Day 1–24 h prior to the test, the animals were habituated (10 min) in an open field arena (no additional objects) to familiarize them with the experimental environment. Day 2 – training session – the animals were placed in the arena with two identical objects (10 min). Day 3–24 h after the training session, a test was performed (to investigate long-term memory processes) by placing the animal in the arena with the familiar and a new object (exploration for 10 min). Due to the innate curiosity of mice, the NOR test does not require lengthy schedules of training sessions or the use of additional reinforcement. Animals should naturally, after recognizing a familiar object, spend more time with the new one. A preference index was used to analyze the results, which is expressed as the amount of time spent recognizing a new object (B), relative to the amount of time spent recognizing a known object (A). With undisturbed cognitive processes, the time spent recognizing a known object (A) is shorter than the time spent recognizing a new object (B).

### The Morris water maze (MWM)

The MWM is one of the most widely used tests to assess spatial memory in rodents. The learning impairments observed in MWM have been shown to be independent of locomotor abnormalities, as locomotor limitations of R6/1 mice do not affect the swimming speed of the animals [44]. The experimental set-up consisted of a black circular of water with a diameter of 150 cm and a depth of 60 cm. The experiment was recorded using a black and white analogue camera connected to a processing device (EthoVision XT, Noldus, Netherlands) which optically divided the pool into four equal parts and recorded the time the animal spent in each quadrant. The pool was equipped with a contrasting (landmark) point on the wall (above the water surface) and a circular platform (made of transparent plastic) with a diameter of 10 cm, placed centrally in the area of one of the quadrants (CQ). The experiment was conducted for five consecutive days at the same time of day (from 8:00 to 12:00 a.m.), according to the previously described procedure [45]. Briefly, each time, milk was added to the water to increase the contrast between the animal and the pool, and to enable its movement to be recorded. The following procedures were conducted on consecutive days. Day 1 – training session – the mouse was placed in the water, with its head facing the pool wall in the quadrant opposite the platform. During the training session, the transparent platform was placed 1 cm above the water surface to allow the animal to consciously remember its location. When the mouse did not find the safe point (platform) within the set time of 60 s, it was gently, manually directed to the platform, where it remained for 10 s, before being transferred to the home cage. When the mouse found the platform in less than 60 s, it remained on the platform for an additional 5 s until the recording was completed. Days 2–4 – spatial memory test – the platform was placed in the pool about 1 cm below the water surface, centrally in the same quadrant (CQ) as for the training session, in such a way that it became directly invisible to the animal. The experiment was started by placing the mouse in the water in the same way as during the training session. During each session, the mouse swam in the water until it found a safe place (latency) – entering the flat surface of the platform – or until 120 s elapsed. When the mouse did not find the platform within the set time, it was manually guided to locate it and remained on the platform for a further 10 s. During the experiment, the following parameters were recorded to assess the progress of spatial memory processes: a reduction in the distance covered, an increase in the amount of time spent in the CQ, and a reduction in the time required to find the platform (latency). Day 5 – the platform was removed from the pool (probe test). The experiment started by placing the mouse in the same quadrant as on the previous days, facing the pool wall. Recordings were made for 60 s. In order to assess the correctness of the memory processes, the time to reach CQ in which the platform was located in previous repetitions, was tested. Each animal was subjected to one experimental session on a particular day, consisting of four repetitions, separated by a 10-min interval. The test was performed three times (when the mice reached 9, 18, and 26 weeks of age). The results are presented as an average of each testing day, not including the training session.

### Measurement of locomotor activity in actometers

The locomotor activity level of the animals was assessed in actometers (Opto Varimex Minor-Columbus, USA). An actometer consists of four plexiglass walls (43 × 43 × 20 cm). At the moment of movement, infrared beams are sent out via a photocell, and then they are counted by a digital counter. The movements analyzed in this test are divided into horizontal (movements in the horizontal plane), vertical (movements in the vertical plane), and ambulatory (such as when an animal cleans its body). The test was conducted at a fixed time between 2:00 p.m. and 3:00 p.m. and lasted 10 min. The test was performed three times (when the mice reached 9, 18, and 26 weeks of age).

### The open-field test

The open-field test is designed to assess the stress-sensitivity of animals, thanks to the stress factor of an open, bright space. In addition, the large space allows the locomotor activity of animals to be measured. The test was conducted in a wooden, white field, bounded by walls (100 × 100 × 60 cm), where the floor was divided into 25 squares (5 × 5 squares), forming the inner area (central squares, located inside the whole area) and the outer area (peripheral squares, located at the border of the whole area). Recording was carried out for 15 min using EthoVision XT 10 software (Noldus, Wageningen, the Netherlands). The experiments were performed at a fixed time, between 2:00 and 3:00 p.m. Animals were placed individually, always facing the corner of the open field. After the test was completed, the animals were placed in their home cages. Between each recording, the field was washed with 70% ethanol solution and allowed to dry for 5 min, in order to minimize the effect of odor stimuli on the reactions studied. The test was performed three times (when the mice reached 9, 18 and 26 weeks of age). Responses considered during the experiment were as follows: time spent in inner and outer areas (central and peripheral squares, respectively), frequency of entry into inner and outer areas, distance traveled in inner and outer areas, velocity, exploration time, and immobility.

### The Rota-rod test

Motor coordination was assessed using a rota-rod apparatus (Yamato Instruments Corporation, India). A mouse was placed on a stationary tube (0 rpm) for three successful trials, then for another three trials at 3 rpm for 120 s. The speed was then increased from 3 to 30 rpm for 300 s at a constant rate. The speed of 30 rpm was maintained for 120 s. The rod was rotated in a clockwise direction. The time in which the mouse manages to maintain its balance on the rod was measured. Recording was preceded by habituating the animals to the test conditions and determining the initial parameters which were an indicator of the current level of motor coordination of the animals. The essential measurement was conducted three times, when the mice reached 9, 18, and 26 weeks of age.

### The grip strength measurement

Grip strength was measured using a computerized grip-force-meter (Model 47200, Ugo-Basile, Varese, Italy). The apparatus consisted of a metal rod in the shape of a “T”, connected to a force transducer. To measure the grip strength of a mouse’s hind paws, the animal was gently held by the base of its tail, allowing the animal to grasp the metal rod using its hind paws. To prevent the mouse from grasping the rod with its front paws while straining, the animal was allowed to grasp the wire cylindrical mesh first. As soon as the mouse grabbed the metal rod, connected to the force transducer, the mouse was pulled backwards by its tail until it lost its grip. The maximum grip force was automatically recorded in the instrument in the form of grams (g). The test was conducted in triplicate for each animal. The essential measurement was conducted three times, when the mice reached 9, 18, and 26 weeks of age.

### Wire handling test

A wooden rod was placed 30 cm above the ground, the measurement included the time the mouse stayed on the rod after it was suspended with its two front paws in a fixed position with its head facing the experimenter. Recording was started when the animal was exactly in the middle of the bar. In addition, the time the animal walked along the rod, from the starting point to the target point, at the end of which the home cage was placed, was measured. The odor stimulus of the familiar home environment stimulated the animal to complete the task faster. The essential measurement was conducted three times, when the mice reached 9, 18, and 26 weeks of age.

### Blood collection

The procedure was performed under anesthesia (a mixture of ketamine (87.5 mg/kg) and xylazine (12.5 mg/kg) at the dose of 100 µl/mouse). Blood was drawn from the venous plexus inside the orbit behind the eyeball, using a capillary sputtered with EDTA. It was then placed in a tube with the same anticoagulant. The procedure was performed 3 times during the experiment to study the correlation of changes in blood parameters with changes in behavior. The last collection of approximately 0.5–1.0 ml was performed postmortem by intracardiac puncture under isoflurane anesthesia (2.5%, flow rate 0.5 l/min; Bitmos OXY 6000 pump, Bitmos GmbH, Düsseldorf, Germany). Next, hematological parameters were analyzed using the Horiba ABX, Micros ES 60 analyzer (Horiba Medical, Japan). The following parameters were measured in the white blood cell system: the absolute number of leukocytes, monocytes, and granulocytes and the percentage ratio of the study population (lymphocytes, monocytes, and granulocytes) to the total leukocyte count. In the red blood cell, the following parameters were examined: hemoglobin (HGB), hematocrit (HCT), mean red blood cell volume (MCV), mean hemoglobin level (MCH), mean hemoglobin concentration (MCHC), and platelet count (PLT). The remaining peripheral blood was centrifuged for 10 min (400 × g) at 4°C to isolate the plasma. The plasma was then stored in a deep freezer (−80°C) until further analysis.

### Measurement of concentrations of cytokines and hormones

Plasma IL6, TNF, IL1B, IL10, and corticosterone concentrations were determined by enzyme-linked immunoassay (ELISA) using commercially available kits (My BioSource Inc., MBS730957, MBS825075, MBS2021142, MBS2023278, respectively) according to the manufacturer’s instructions, and using the EnSpire Multimode Plate Reader (PerkinElmer, Waltham, USA) system set to 450 nm. The cytokine and hormone concentrations were calculated based on the standard curve generated by the EnSpire Software, based on the absorbance of standard samples.

### Euthanasia

When all behavioral, immunological, and biochemical analyses were completed, the animals were anesthetized with isoflurane inhalation anesthesia (2.5%, flow rate 0.5 l/min). Then, mice were given a lethal intraperitoneal dose of pentobarbital at 120 mg/kg.

### Muscle collection

Quadriceps thigh muscles were taken from both hind limbs, and the muscles were cleaned of fat and connective tissue. The muscles thus isolated were placed in tubes, which were then frozen in liquid nitrogen and stored at −80°C until further procedures. The muscles were homogenized in a Bullet BlenderTM (Next Advance, 4116-BBY24M-CE). Muscle tissue was cut into pieces, weighing about 100 mg, and placed in a microcentrifuge tube. Zirconium silicate beads (0.5 mm; Next Advance Inc., ZROB05) of 300 mg were added to the tube. Then, 0.2 ml of the homogenization buffer (T-PER Tissue Protein Extraction Reagent; Thermo Scientific 78,510) was added. The tube was sealed and placed in a Bullet BlenderTM, the speed was set to 9, while the time was set to 3 min. The homogenate was used for the determination of reactive oxygen species and CK levels.

### Myocardial collection

The heart muscle was cut into pieces ranging from 5 to 300 mg and then placed in a microcentrifuge tube. Stainless steel beads with a diameter of 1.6 mm (Next Advance Inc., SSB16) were added to the tube. The mass of the beads had to be equal to the mass of the tissue in the tube. Then, T-PER Tissue Protein Extraction Reagent was added in a volume ratio of 2:1 to the volume of the tissue (max. 0.6 ml of the homogenization buffer). The tube was sealed and placed in a Bullet BlenderTM, the speed was set to 8, and the time was set to 4 min. The homogenate was used to determine TNN levels.

### Determination of reactive oxygen species (ROS), TNN (troponin I) and CK levels

The levels of ROS, TNN, and CK were determined by enzyme-linked immunoassay (ELISA), using commercially available kits (My BioSource Inc., MBS2803510, MBS2104865, and MBS9301010, respectively), according to the manufacturer’s instructions and using the EnSpire Multimode Plate Reader system, set to 450 nm. The concentration of analyzed compounds was calculated employing the standard curve, generated by EnSpire Software, based on the absorbance of standard samples.

### Genistein

Genistein (4′,5,7-trihydroxyisoflavone or 5,7-dihydroxy-3-(4-hydroxyphenyl)-4 H–1-benzopyran-4-one) was purchased from Pharmaceutical Research Institute in Warsaw (99% purity; 446-72-0). Stock solutions were prepared in DMSO to obtain 30, 60, and 100 µM genistein; they were stored at −20°C.

### Antibodies

The following antibodies were used, anti-TFEB rabbit antibody (Cell Signaling Technology, D207D), anti-RPS6KB/S6K rabbit antibody (Cell Signaling Technology, 9202), anti-phospho-RPS6KB/p70 S6 kinase (Thr389) rabbit antibody (Cell Signaling Technology, 9205), anti-EIF4EBP1 rabbit antibody (Cell Signaling Technology, 9452), and anti-phospho-IEF4EBP1 (Thr37/46) rabbit antibody (Cell Signaling Technology 9459), mouse anti-human HTT/huntingtin clone 1HU-4C8 (Sigma-Aldrich, MAB2166), anti-LC3 antibody (MBL International, PM036), rabbit anti-FOXO3/FOXO3a for immunohistochemistry (Cell Signaling Technology 12,829), monoclonal anti-FOXO3 for western blotting (Cell Signaling Technology 99,199), and monoclonal anti-phosphorylated FOXO3 for western blotting (Cell Signaling Technology, 8174). The anti-mouse and anti-rabbit antibodies, as well as anti-GAPDH antibodies, were from Sigma Aldrich (A9044, A0545, and G9295, respectively).

### Cell lines and cell cultures

Lines of fibroblasts derived from four HD patients and four age- and sex-matched controls were used in this work; these lines were characterized and described in detail previously [46,47]. Both HD and control fibroblasts were obtained from males only, and the age range was 41–54 and 43–51 years, respectively. Cells were cultured on 10-cm plates in DMEM medium (Thermo Fisher Scientific Inc., 11-995-065), supplemented with 10% FBS (Thermo Fisher Scientific Inc., 10270–106) and 1% antibiotic/antimycotic solution (Sigma-Aldrich Co. LLC., 15240–062). A temperature of 37°C and a humidified atmosphere of 95% air/5% CO_2_ were employed as standard culture conditions.

### Silencing of the expression of the *FOXO3* gene

The siRNA-mediated silencing of gene expression in human fibroblasts was performed according to the previously described procedure [48] while minor modifications. The Lipofectamine 3000 transfection reagent (Thermo Fisher Scientific, L3000001) was used for transfection, according to the manufacturer’s instructions. Two siRNAs specific for the human *FOXO3* gene were used; the validated oligonucleotides vhs41092 and vhs41096 were purchased from Thermo Fisher Scientific (1299001). Following 48-h incubation, the silencing efficiency, determined by RT-qPCR, was at the level of 70–80% which corresponded to the previously achieved levels [21].

### Immunoblotting

6 × 10^5^ HD or control fibroblasts were passaged on plates of 10-cm diameter, and they were allowed to attach overnight. Fibroblasts were treated with either 0.1% DMSO (final concentration) (in control experiments) or 30, 60, and 100 µM genistein for 24 h. Cell lysis was conducted with a solution containing 1% Triton X-100 (Sigma Aldrich, 9002-93-1), 0.5 mM EDTA, 150 mM NaCl, 50 mM Tris, pH 7.5, and a mixture of protease and phosphatase inhibitors (Roche Applied Science 05,892,791,001 and 11,873,580,001). Following centrifugation in a microfuge at 3,000 × g for 10 min, supernatants were used for protein separation. In most experiments, proteins were separated employing the WES system (WES – Automated Western Blots with Simple Western; ProteinSimple, San Jose, California, USA), with 12–230 kDa Separation Module (SM-W003) and detected with Anti-Mouse Detection Module (DM-002) or Anti-Rabbit Detection Module (DM-001), according to the manufacturer’s instruction. The total protein level, determined using a Total Protein Detection Module for Chemiluminescence (DM-TP01), was used as the loading control. Since capillary electrophoresis (used in the WES system) does not allow the separation of two forms of the LC3 protein (LC3-I and LC3-II) [49], regular western blotting was used when investigating this autophagy marker. Briefly, following clearance of cell lysates by centrifugation, polypeptides were separated by SDS-PAGE and then transferred to a PVDF membrane. The membrane was blocked with 5% nonfat dry milk in PBST (Cepham Life Sciences 10,621) buffer, and incubated with primary antibodies overnight at 4°C. Then, the membrane was incubated with secondary antibodies at room temperature for 1 h. To detect a specific signal, a solution of substrates for HRP (BIO-RAD, 170–5061) was added, and the membrane was exposed to the X-Ray film. The intensities of bands were analyzed with the QuantityOne software.

### Fluorescence microscopy

Fibroblasts (4 × 10^4^ cells) were passaged on coverslips in 6-well plates and allowed to attach overnight. Cells were treated with either 0.1% DMSO (controls), or 30, 60 and 100 µM genistein for 24 h. The cells were fixed with 2% paraformaldehyde in phosphate-buffered saline (PBS; Merck 806,552) and rinsed with 0.1% Triton X-100 in PBS. Then, the samples were blocked with 5% BSA (Sigma Aldrich, A7906) and 1.5% glycine in PBS for 1 h. Following overnight incubation with primary antibody (1:1000) dissolved in PBS, the cells were rinsed with PBS three times, and incubated with secondary antibodies dissolved in PBS (1:4000) for 2 h. After washing with PBS (5 times) coverslips were affixed to glass slides with a mounting medium. LysoTracker Red was purchased from Life Technologies (L-7528). The next day, the samples were analyzed using the Leica (Germany) fluorescence microscope.

### Statistical analysis

The results are presented as mean values ± standard deviation (SD). For statistical analyses of the results, the SPSS 21.0 (SPSS Inc., Armonk, USA) software was used. The normality of the distribution of variables was checked with the Kolmogorov-Smirnov test and the homogeneity of the variances with the Levene test. When the outcome of the Kolmogorov-Smirnov test indicated that the data were not distributed normally, non-parametric Kruskal-Wallis and Dunn tests were used for further analysis. For other parameters, two-way ANOVA and Tukey’s post hoc tests were performed. The *p* value lower than 0.05 was considered statistically significant.

## Supplementary Material

SupplementaryAutophagyHDR6.docx

## Data Availability

Raw data and all materials are available from the authors at request.
