# Supplementary material for: Correction of symptoms of Huntington disease by genistein through FOXO3-mediated autophagy stimulation
Source: Autophagy. 2023 Nov 22;20(5):1159–82. doi: 10.1080/15548627.2023.2286116 (PMC11135876; doi:10.1080/15548627.2023.2286116)
Supplement: SupplementaryAutophagyHDR6.docx [file KAUP_A_2286116_SM3455.docx]

**Supplementary Material**

**
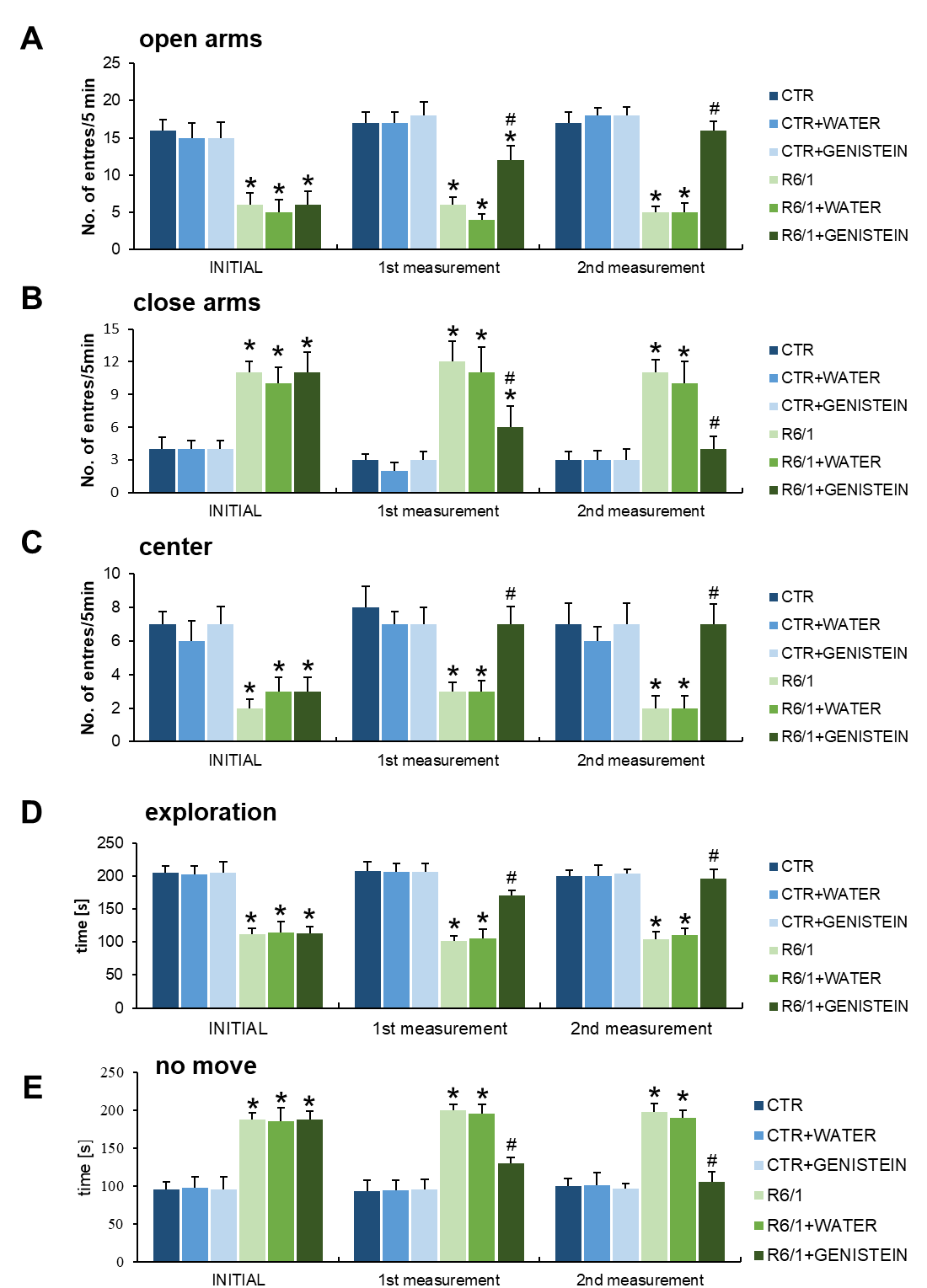
**

**Figure S1.** Correction of cognitive impairments and anxiety behavior in HD mice by genistein as assessed in the elevated plus-maze (EPM) test. HD mice (the R6/1 model) or control animals (the C57BL/6J line) were either untreated, treated with water, or treated with genistein (at the final dose of 150 mg/kg/day), starting from the age of 16 weeks. The tests were performed with mice at the age of 9, 18 and 26 weeks (marked as INITIAL, 1^st^ measurement, and 2^nd^ measurement, respectively). Results are shown as mean values from measurements performed with 6 mice in each group with error bars indicating SD. Statistically significant differences (at *p*<0.05) relative to untreated control (CTR) mice and HD mice (the R6/1 line) are indicated by asterisks and hashtags, respectively.


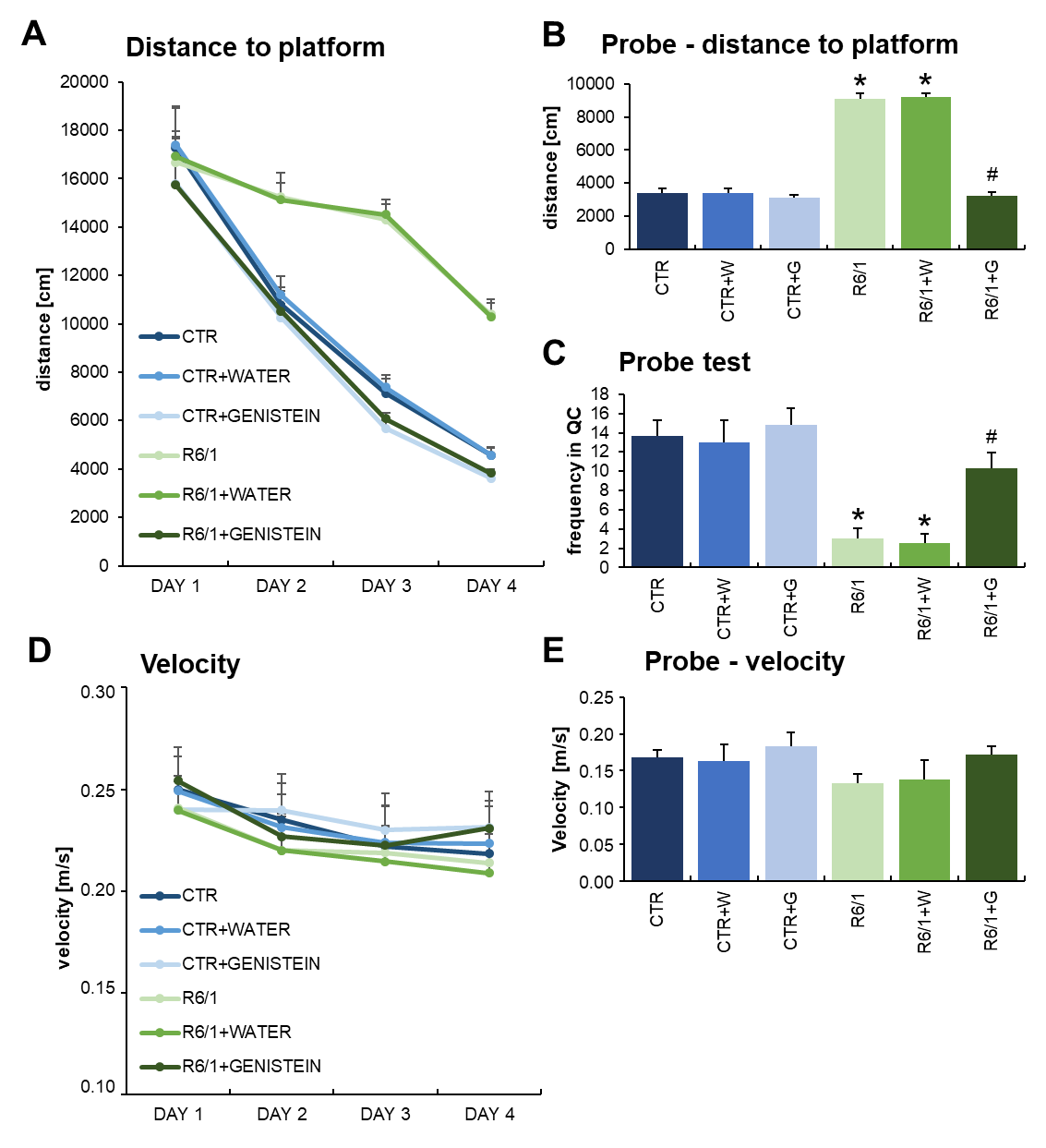


**Figure S2.** Correction of cognitive impairments in HD mice by genistein as assessed in the Morris water maze (MWM) test. HD mice (the R6/1 model) or control animals (the C57BL/6J line) were either untreated, treated with water, or treated with genistein (at the final dose of 150 mg/kg/day), starting from the age of 16 weeks. Results are shown as mean values from measurements performed with 6 mice in each group with error bars indicating SD. Statistically significant differences (at *p*<0.05) relative to untreated control (CTR) mice and HD mice (the R6/1 line) are indicated by asterisks and hashtags, respectively.


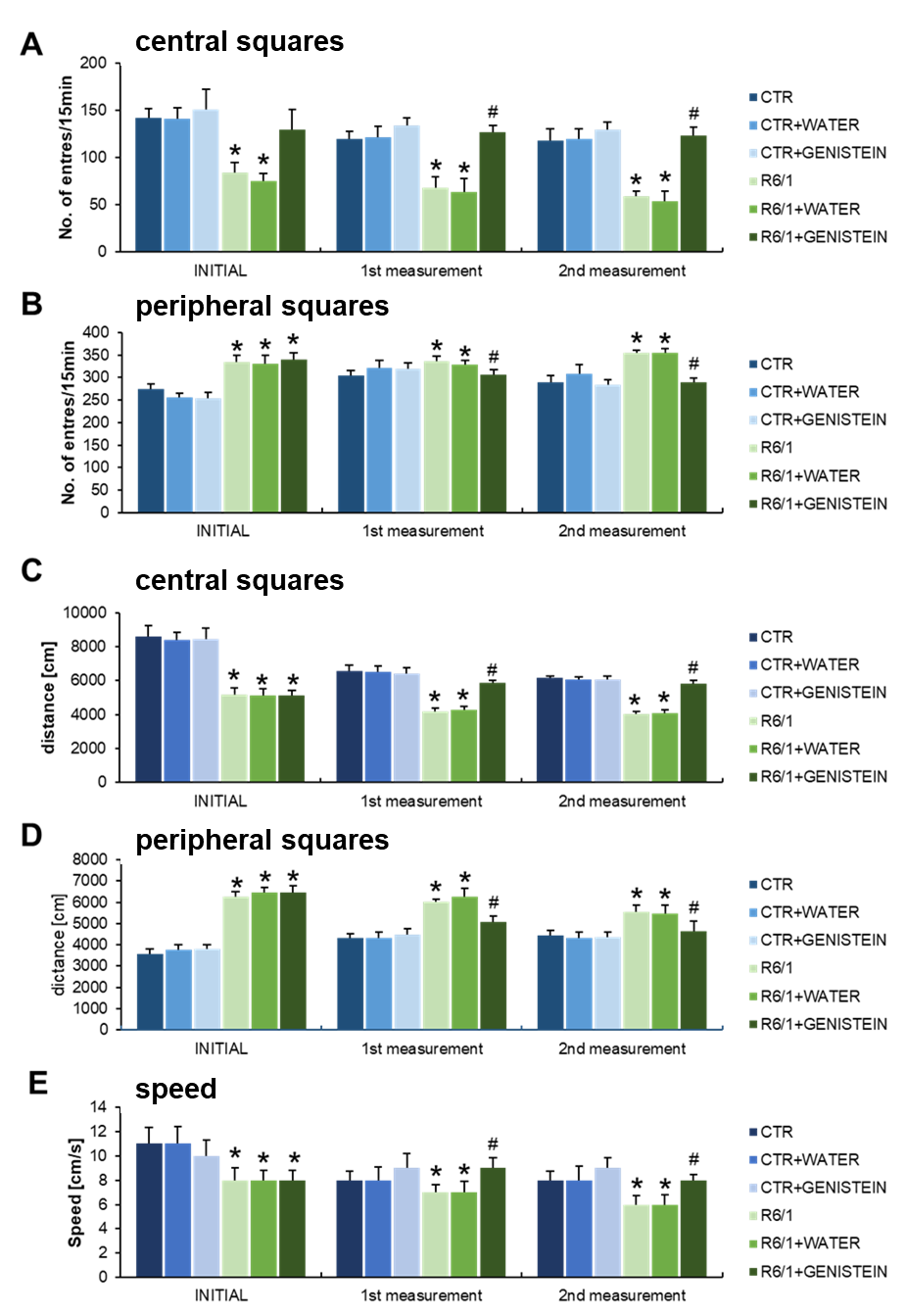


**Figure S3.** Correction of anxiety behavior in HD mice by genistein as assessed in the open field test. HD mice (the R6/1 model) or control animals (the C57BL/6J line) were either untreated, treated with water, or treated with genistein (at the final dose of 150 mg/kg/day), starting from the age of 16 weeks. The tests were performed with mice at the age of 9, 18 and 26 weeks (marked as INITIAL, 1^st^ measurement, and 2^nd^ measurement, respectively). Results are shown as mean values from measurements performed with 6 mice in each group with error bars indicating SD. Statistically significant differences (at *p*<0.05) relative to untreated control (CTR) mice and HD mice (the R6/1 line) are indicated by asterisks and hashtags, respectively.


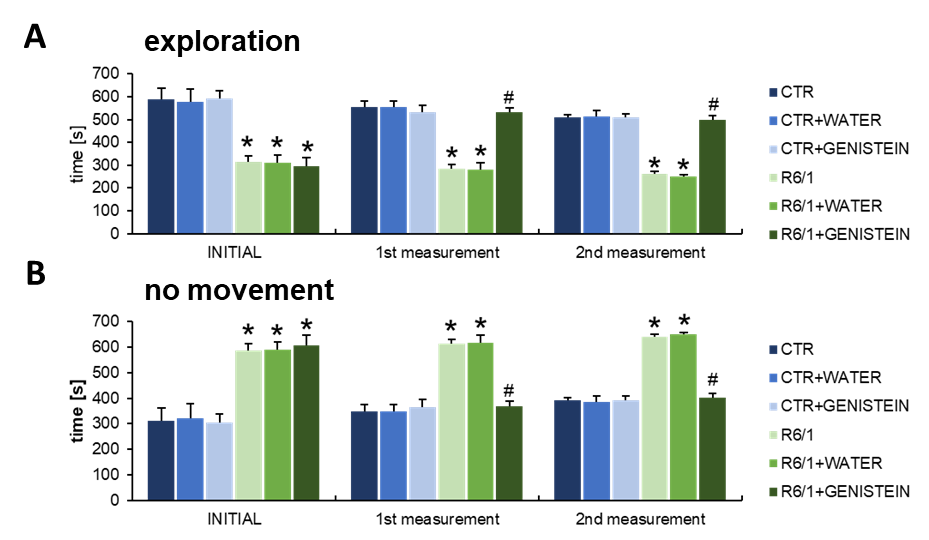


**Figure S4.** Correction of cognitive impairments in HD mice by genistein as assessed in the open field test. HD mice (the R6/1 model) or control animals (the C57BL/6J line) were either untreated, treated with water, or treated with genistein (at the final dose of 150 mg/kg/day), starting from the age of 16 weeks. The tests were performed with mice at the age of 9, 18 and 26 weeks (marked as INITIAL, 1^st^ measurement, and 2^nd^ measurement, respectively). Results are shown as mean values from measurements performed with 6 mice in each group with error bars indicating SD. Statistically significant differences (at *p*<0.05) relative to untreated control (CTR) mice and HD mice (the R6/1 line) are indicated by asterisks and hashtags, respectively.

**
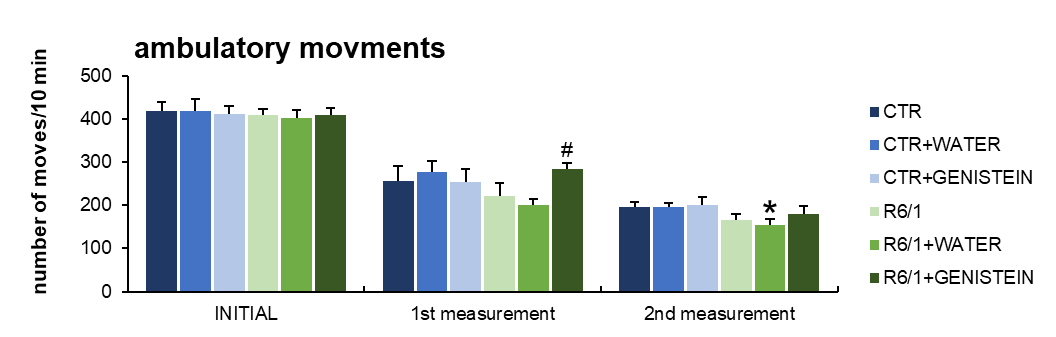
**

**Figure S5.** Ambulatory movements in HD mice by treated with genistein as assessed in an actometer. HD mice (the R6/1 model) or control animals (the C57BL/6J line) were either untreated, treated with water, or treated with genistein (at the final dose of 150 mg/kg/day), starting from the age of 16 weeks. The tests were performed with mice at the age of 9, 18 and 26 weeks (marked as INITIAL, 1^st^ measurement, and 2^nd^ measurement, respectively). Results are shown as mean values from measurements performed with 6 mice in each group with error bars indicating SD. Statistically significant differences (at *p*<0.05) relative to untreated control (CTR) mice and HD mice (the R6/1 line) are indicated by asterisks and hashtags, respectively.


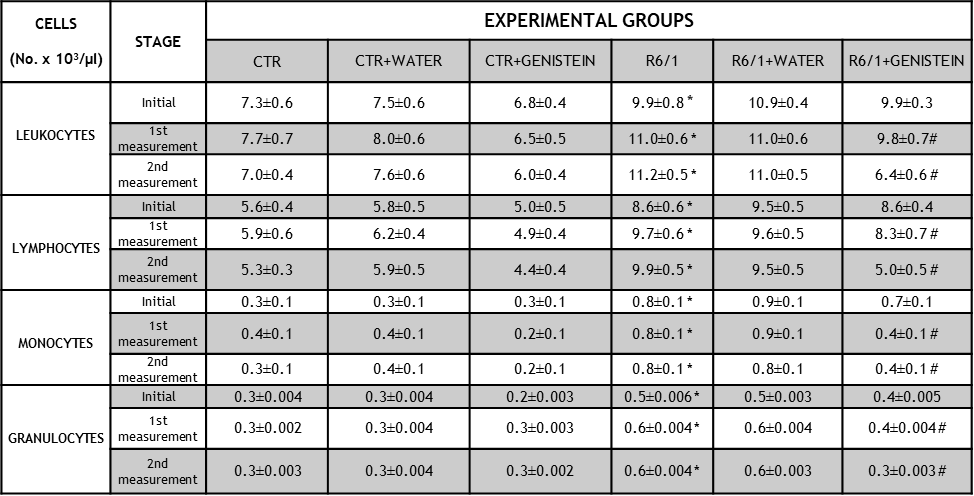


**Figure S6.** Effects of genistein on hematological parameters (number of leukocytes, lymphocytes, monocytes, and granulocytes) in HD mice. HD mice (the R6/1 model) or control animals (the C57BL/6J line) were either untreated, treated with water, or treated with genistein (at the final dose of 150 mg/kg/day), starting from the age of 16 weeks. Results are shown as mean values from measurements performed with 6 mice in each group with error bars indicating SD. Statistically significant differences (at *p*<0.05) relative to untreated control (CTR) mice and HD mice (the R6/1 line) are indicated by asterisks and hashtags, respectively.


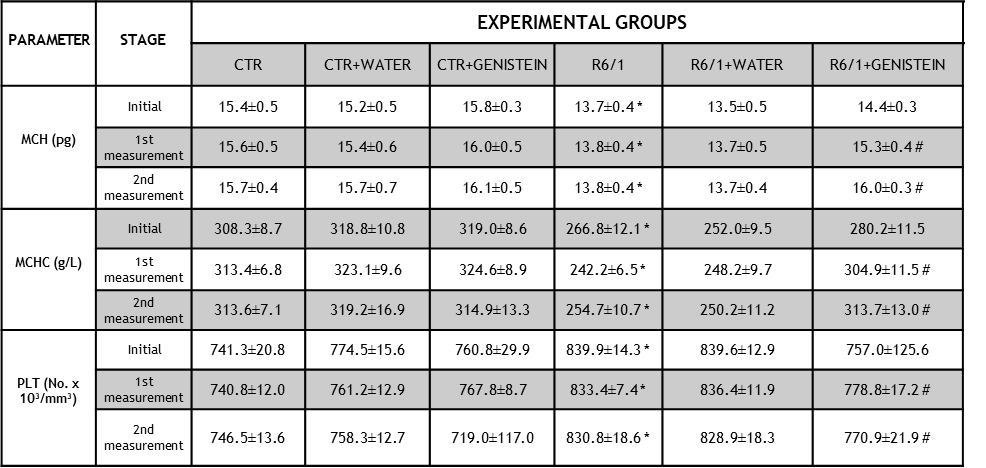


**Figure S7.** Effects of genistein on hematological parameters (MCH, MCHC, and PLT) in HD mice. HD mice (the R6/1 model) or control animals (the C57BL/6J line) were either untreated, treated with water, or treated with genistein (at the final dose of 150 mg/kg/day), starting from the age of 16 weeks. Results are shown as mean values from measurements performed with 6 mice in each group with error bars indicating SD. Statistically significant differences (at *p*<0.05) relative to untreated control (CTR) mice and HD mice (the R6/1 line) are indicated by asterisks and hashtags, respectively.


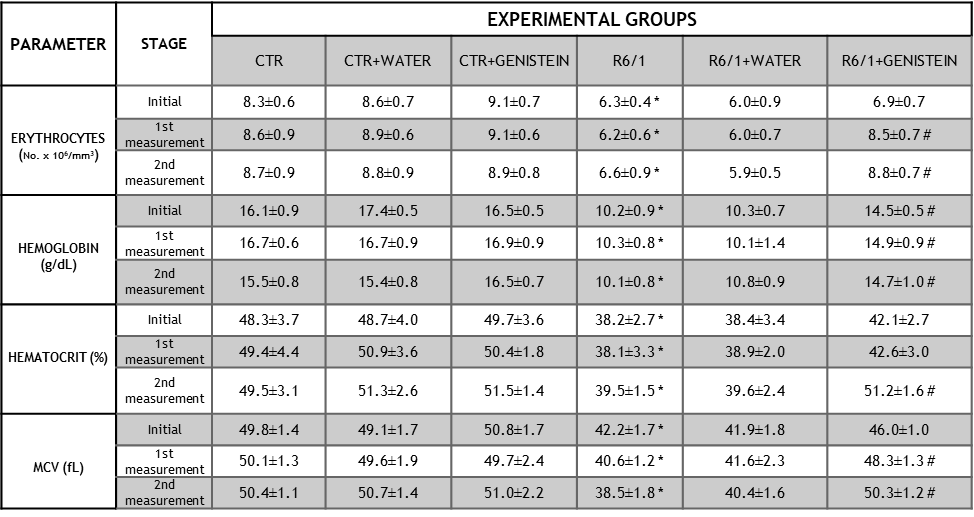


**Figure S8.** Effects of genistein on hematological parameters (number of erythrocytes, level of hemoglobin, hematocrit, and MCV) in HD mice. HD mice (the R6/1 model) or control animals (the C57BL/6J line) were either untreated, treated with water, or treated with genistein (at the final dose of 150 mg/kg/day), starting from the age of 16 weeks. Results are shown as mean values from measurements performed with 6 mice in each group with error bars indicating SD. Statistically significant differences (at *p*<0.05) relative to untreated control (CTR) mice and HD mice (the R6/1 line) are indicated by asterisks and hashtags, respectively.


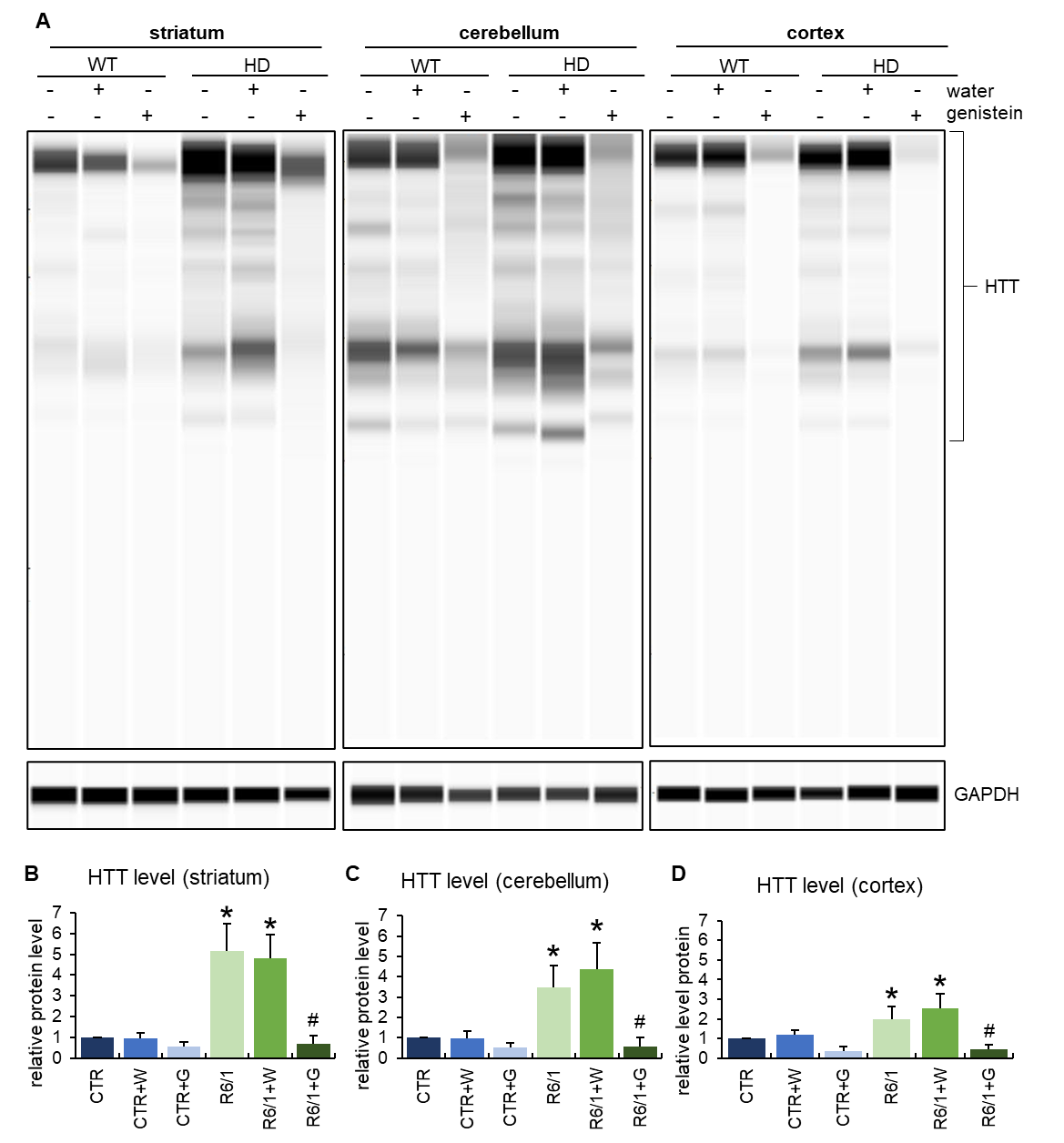


**Figure S9.** Reduction of levels of HTT aggregates in the brains of HD mice treated with genistein, as assessed in western-blotting experiments using an alternative antibody. HD mice (the R6/1 model) or control animals (the C57BL/6J line) were either untreated, treated with water, or treated with genistein (at the final dose of 150 mg/kg/day), starting from the age of 16 weeks. Levels of HTT were measured in striatum, cerebellum, and cortex of brains of mice at the age of 36 weeks. Panel **A** shows representative western blots. Panels **B-D** represent quantification of the results, shown as mean values from measurements performed with 6 mice in each group with error bars indicating SD. Statistically significant differences (at *p*<0.05) relative to untreated control (CTR) mice and HD mice (the R6/1 line) are indicated by asterisks and hashtags, respectively.
